# MicroRNA-23b Promotes Avian Leukosis Virus Subgroup J (ALV-J) Replication by Targeting *IRF1*

**DOI:** 10.1038/srep10294

**Published:** 2015-05-18

**Authors:** Zhenhui Li, Biao Chen, Min Feng, Hongjia Ouyang, Ming Zheng, Qiao Ye, Qinghua Nie, Xiquan Zhang

**Affiliations:** 1 Department of Animal Genetics, Breeding and Reproduction, College of Animal Science, South China Agricultural University, Guangzhou 510642, Guangdong, China; 2 Guangdong Provincial Key Lab of Agro-Animal Genomics and Molecular Breeding and Key Lab of Chicken Genetics, Breeding and Reproduction, Ministry of Agriculture, Guangzhou 510642, Guangdong, China

## Abstract

Avian leukosis virus subgroup J (ALV-J) can cause several different leukemia-like proliferative diseases in the hemopoietic system of chickens. Here, we investigated the transcriptome profiles and miRNA expression profiles of ALV-J-infected and uninfected chicken spleens to identify the genes and miRNAs related to ALV-J invasion. In total, 252 genes and 167 miRNAs were differentially expressed in ALV-J-infected spleens compared to control uninfected spleens. miR-23b expression was up-regulated in ALV-J-infected spleens compared with the control spleens, and transcriptome analysis revealed that the expression of interferon regulatory factor 1 (*IRF1*) was down-regulated in ALV-J-infected spleens compared to uninfected spleens. A dual-luciferase reporter assay showed that *IRF1* was a direct target of miR-23b. miR-23b overexpression significantly (P = 0.0022) decreased *IRF1* mRNA levels and repressed IRF1-3′-UTR reporter activity. *In vitro* experiments revealed that miR-23b overexpression strengthened ALV-J replication, whereas miR-23b loss of function inhibited ALV-J replication. *IRF1* overexpression inhibited ALV-J replication, and *IRF1* knockdown enhanced ALV-J replication. Moreover, *IRF1* overexpression significantly (P = 0.0014) increased *IFN-β* expression. In conclusion, these results suggested that miR-23b may play an important role in ALV-J replication by targeting *IRF1*.

Avian leukosis viruses (ALVs) belong to the genus *Alpharetrovirus* of the *Retroviridae* family. Chicken ALVs can be divided into exogenous (subgroups A, B, C, D and J) and endogenous (subgroup E) viruses based on the mode of their transmission^1^. Since the first report of myeloid leukosis induced by ALV-J in 1988^2^, this disease has become widespread and subsequently led to serious economic losses in poultry production^3^,^4^. Both broilers and layers can be infected by ALV-J, which then induces the formation of various types of tumors, including hemangioma and myelocytoma^2^,^5^. Thus far, the pathogenesis mechanisms of ALV have been explained by three theories: promoter insertion^6^, enhancer activation^7^, and viral oncogenes^8^. However, the genetic mechanisms underlying host resistance to ALV-J infection remain unclear.

Mature microRNAs (miRNAs) are single-stranded non-coding small RNAs of 21-25 nucleotide (nt) lengths that typically reduce the translation and stability of mRNAs. miRNA deregulation has been shown to play pivotal roles in tumorigenesis and progression via the up-regulation of oncogenes and the silencing of tumor suppressor genes, respectively^9^,^10^. For example, miR-29c in hepatocellular carcinoma and miR-296-5p in prostate cancer function as tumor suppressors^11^,^12^. In contrast, miR-135b and the miR-17-92 cluster act as oncogenes in colon cancer^13^ and in malignant lymphoma^14^, respectively. In ALV-J-induced tumors, the aberrant expression of miRNAs such as miR-221, miR-375 and miR-1650 contributes to tumor cell growth, apoptosis, migration and invasion by targeting genes involved in those cellular pathways^15^,^16^,^17^. However, thus far, no miRNA involved in the regulation of the host anti-ALV-J response has been elucidated.

The objective of the present study was to investigate the genetic basis of host resistance against ALV-J and to identify key miRNAs and target genes responsible for the host anti-ALV-J response. In the current study, the transcriptome profiles and miRNA expression profiles of ALV-J-infected and uninfected chicken spleens were scanned to identify genes and miRNAs related to ALV-J invasion. Then, targets of these differentially expressed miRNAs were predicted, and differentially expressed genes (DEGs) that are the targets of the differentially expressed miRNAs were selected. Negatively correlated miRNA-gene pairs were used for further miRNA-target and target-target interaction network analysis and GO analysis. Subsequently, *in vitro* experiments were performed to identify candidate genes and miRNAs involved in host anti-ALV-J mechanisms.

## Results

### Virus isolation and ALV-J infection identification

**Figure 1:**
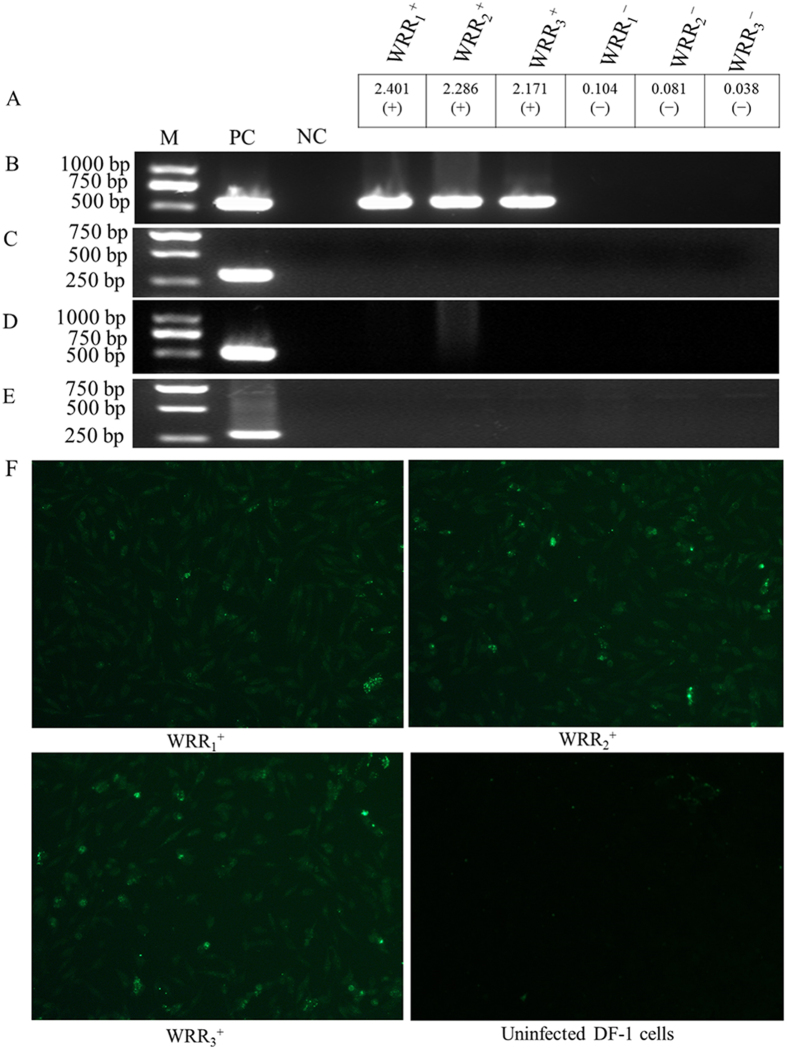
Virus isolation and identification in DF-1 cells by ELISA, PCR and IFA. DL2000 was used as the DNA marker (M) (TaKaRa). Uninfected DF-1 cells were used as the negative control (NC). (**A**, **B**) DF-1 cells infected with ALV-J were used as the positive control (PC) for ELISA and for PCR with the primer pair H5/H7. (**C**) DF-1 cells infected with Rous-associated virus type 1 (subgroup A) served as the positive control (PC) for PCR with the primer pair H5/AD1. (**D**) DNA samples infected with MDV served as the positive control (PC) for PCR with the primer pair MDV-F/MDV-R. (**E**) DNA samples infected with REV served as the positive control (PC) for PCR with the primer pair REV-F/REV-R. (**F**) The IFA results showed ALV-J-specific green fluorescence in WRR_1_^+^, WRR_2_^+^, and WRR_3_^+^at 150x. The full-length gels with ALV-J, exogenous ALVs (A-D), MDV and REV detection are presented in Supplementary Fig. S2 A-D, respectively.

Based on clinical symptoms, 46 chickens were chosen for virus identification. After infected DF-1 cells were incubated for 7 days, 41% (19/46) of these cells were demonstrated to be positive by ELISA. Three ELISA-positive samples (WRR_1_^+^, WRR_2_^+^, WRR_3_^+^) with the highest S/P ratios and three other ELISA-negative samples (WRR_1_^−^, WRR_2_^−^, WRR_3_^−^) with the lowest S/P ratios (Fig. 1A) were chosen for further validation by PCR and indirect immunofluorescence assay (IFA). The PCR products of the DNA extracted from ELISA-positive samples (WRR_1_^+^, WRR_2_^+^, WRR_3_^+^) were tested for positivity using the H5 and H7 primers, whereas no specific products were amplified from the ELISA-negative samples (WRR_1_^−^, WRR_2_^−^, WRR_3_^−^) (Fig. 1B). These same DNA samples did not produce any specific products during PCR with primers used for the detection of other viruses, including exogenous ALVs (Fig. 1C), Marek’s disease virus (MDV) (Fig. 1D) and reticuloendotheliosis virus (REV) (Fig. 1E). IFAs indicated positive results for the ELISA-positive samples (WRR_1_^+^, WRR_2_^+^, WRR_3_^+^) (Fig. 1F) but negative results for the ELISA-negative samples (WRR_1_^−^, WRR_2_^−^, WRR_3_^−^) (data not shown), confirming that the samples WRR_1_^+^, WRR_2_^+^, and WRR_3_^+^were infected with ALV-J, whereas the samples WRR_1_^−^, WRR_2_^−^, and WRR_3_^−^ were not infected.

### Overview of small RNA sequencing

**Figure 2:**
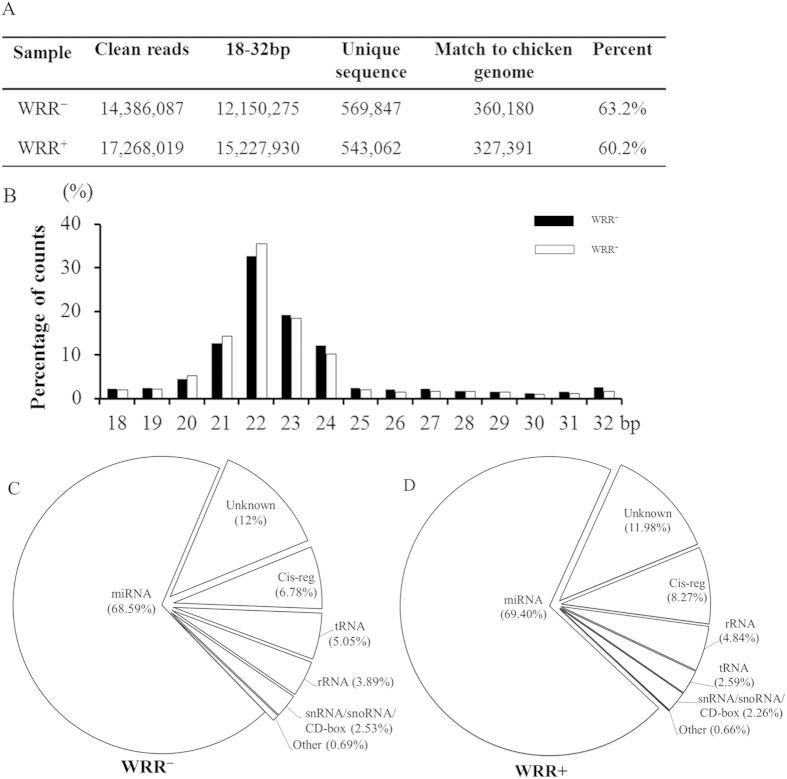
Small RNA sequencing data, size distribution and annotation. (**A**) Numbers of small RNA reads from the WRR^−^ and WRR^+^libraries. (**B**) Length distribution of sequenced small RNAs. (**C**, **D**) Annotations of sequenced small RNAs.

Illumina deep sequencing was used to profile miRNAs expressed in ALV-J-infected and uninfected chicken spleens. After the raw data were filtered, 12,150,275 and 15,227,930 reads of 18-32 bp, which represented 569,847 and 543,062 unique sequences, were obtained from the WRR^−^ and WRR^+^libraries, respectively. By BLAST searching the chicken reference genome, 360,180 WRR^−^ sequences and 327,391 WRR^+^sequences, which accounted for more than 60% of unique sequences, were matched perfectly (Fig. 2A). The length distribution analysis revealed that 22 nt was the most frequent size class among the small RNA sequences, followed by 21 and 23 nt (Fig. 2B). These data were consistent with the size distribution of miRNAs. miRNAs accounted for more than 68% of all clean reads in the WRR^−^ and WRR^+^libraries (Fig. 2C, D).

### Differentially expressed miRNAs

In this study, 476 miRNAs were identified after comparing the unique sequences against chicken miRNA precursors in miRBase 18.0. Based on the matched counts of unique sequences, 167 differentially expressed miRNAs were identified with the DEGseq package using a Benjamini q-value of 0.001 as the cut-off (Supplementary Table S1). In ALV-J-infected spleens, 83 miRNAs exhibited up-regulated expression and 84 exhibited down-regulated expression compared to uninfected samples.

### Overview of transcriptome sequencing

**Figure 3:**
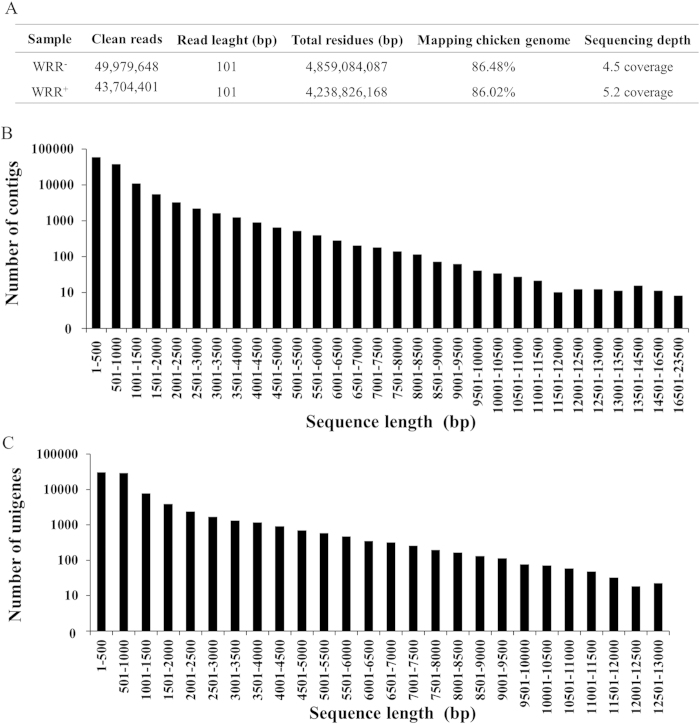
Transcriptome sequencing data and contig and unigene length distributions. (**A**) Draft reads of Illumina deep sequencing. (**B**) The sequence length distribution of contigs. (**C**) The sequence length distribution of unigenes.

When the raw data were processed, 49,979,648 and 43,704,401 clean reads with an average length of 101 bp, which represented total residues of 4,859,084,087 and 4,238,826,168 bp, were obtained from the WRR^−^ and WRR^+^libraries, respectively (Fig. 3A). Subsequently, the clean reads in the two libraries were assembled. Altogether, 121,493 contigs were assembled with an average length of 927 bp (ranging from 300 bp to 23,402 bp), leading to the generation of 82,829 unigenes (Fig. 3B). The lengths of the unigenes varied from 351 bp to 28,928 bp, with an average length of 1,155 bp (Fig. 3C).

### Identification of DEGs

Based on the FPKM value of each gene, 252 DEGs were identified with the DEGseq package using a Benjamini q-value of 0.05 as the cut-off (Supplementary Table S2). In ALV-J-infected spleens, 90 genes were up-regulated and 162 were down-regulated compared to uninfected samples.

### Target prediction and miRNA-mRNA interaction network analysis

To analyze the miRNA transcriptome profile and mRNA transcriptome profile systematically to narrow the field of candidate targets for differentially expressed miRNAs, the differentially expressed genes that were also the targets of the differentially expressed miRNAs were selected. Based on this criterion, 559 miRNA-gene pairs were identified, including 300 negatively and 259 positively correlated pairs (Supplementary Table S3).

**Figure 4:**
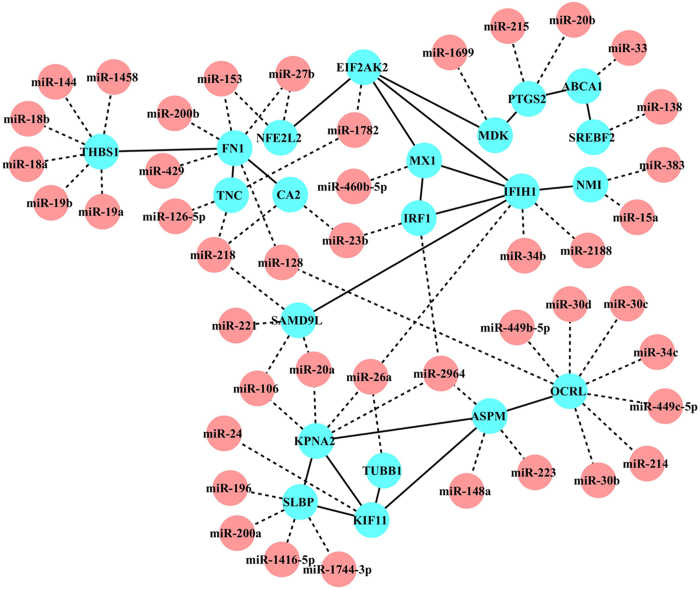
Interaction network of differentially expressed miRNAs and genes. In the regulation network, miRNAs are displayed as red circles, and targets are displayed as blue nodes. Solid lines represent target-target interactions, and dashed lines represent miRNA-target interactions.

Because many studies have demonstrated that miRNAs can provoke mRNA degradation or inhibit mRNA translation^18^, we chose differentially expressed miRNA targets whose gene expression levels were opposite to those of their corresponding miRNAs. Using this principle, 111 genes among the 300 negatively correlated miRNA-gene pairs were selected and further used for target-target network construction. After eliminating the targets that had no relevance to others, a regulatory network involving 44 miRNAs (red nodes) and 21 targets (blue nodes) was generated (Fig. 4). Among the predicted miRNA-target relationships, correlations between miR-18a, miR-19a and the target *THBS1* are reported to be involved in angiosarcomas^19^, while the correlation between miR-200b and target *FN1* is associated with ovarian cancer^20^. Notably, in the target-target interaction network, some genes, including *IRF1*, *MX1*, *NMI* and *IFIH1*, may play essential roles in host immune processes (Table 1). Moreover, these genes could target each other directly or indirectly. Thus, the predicted regulatory network in this study provided several candidates for future studies exploring miRNA-target functions in the control of the host anti-ALV-J response.

**Figure 5:**
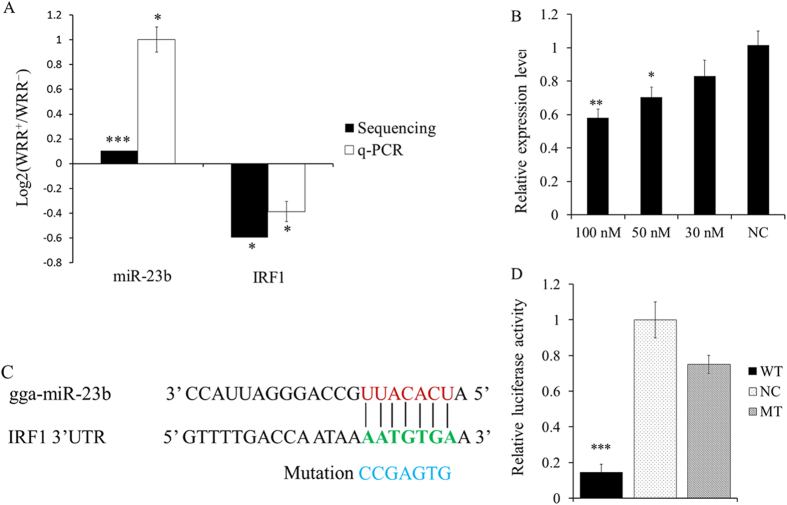
Validation of IRF1 as a direct target of miR-23b. WRR^+^: ALV-J-infected spleens; WRR^−^: uninfected chicken spleens. (**A**) Deep sequencing found that miR-23b was up-regulated and that its corresponding target *IRF1* was down-regulated in ALV-J-infected chicken spleens. The opposing expression patterns of miR-23b and *IRF1* in ALV-J-infected and uninfected samples were confirmed by qPCR. (**B**) In total, 100 nM, 50 nM and 30 nM of miR-23b mimic could decrease *IRF1* expression levels in DF-1 cells at 48 h after transfection. (**C**) miR-23b binding site in the 3′ UTR of chicken *IRF1* mRNA (green). The mutation sequence in the miR-23b binding site is highlighted in blue. (**D**) Dual-luciferase reporter assay of DF-1 cells after co-transfection with wild-type (WT) or mutant 3′ UTR *IRF1* (MT) or negative control plasmid (NC) and miR-23b mimics. Data are presented as means ± S.E.M. of three independent experiments. *, **, and *** indicate P-value significance at the threshold levels of 0.05, 0.01 and 0.001, respectively.

GO functional annotation of these 111 genes is shown in supplementary Fig. S1. Importantly, GO analysis revealed that *IRF1* is associated with many immune-related GO terms, including defense response, regulation of regulatory T cell differentiation, immune effector process and response to virus (Supplementary Table S4). *IRF1*, which was the first identified member of the interferon regulatory transcription factor family, can activate the expression of the *IFNβ* gene, which possesses antineoplastic activity by driving the expression of genes related to anti-viral, anti-proliferative and proapoptotic functions^21^,^22^. In addition, *IRF1* can also recruit the coactivator p300 to induce the expression of *p53*, which functions as a potent tumor suppressor^23^. Previous studies have demonstrated that miR-383^24^ and miR-23a^25^ are regulatory factors for *IRF1*. Network analysis showed that *IRF1* is also a target of gga-miR-23b and gga-miR-2964 (Fig. 4). miR-23b plays a significant role in tumorigenesis^26^ and inhibits virus infection^27^. Interestingly, our analysis showed that miR-23b and its putative target *IRF1* had opposing expression patterns in uninfected samples and ALV-J-infected samples (Fig. 5A). Therefore, miRNA-target interactions between miR-23b and the *IRF1* gene can be considered candidates for further study.

### The 3′ UTR of *IRF1* is directly targeted by miR-23b

To validate the target relationship between miR-23b and *IRF1*, a dual-luciferase reporter gene assay was conducted in DF-1 cells. The transfection efficiency analysis showed that 50 nM and 100 nM of a miR-23b mimic could significantly (P = 0.0189) and highly significantly (P = 0.0022) decrease *IRF1* expression levels in DF-1 cells at 48 h after transfection, respectively (Fig. 5B). Therefore, we selected 100 nM as the best concentration for the following experiments. The miR-23b mature sequence and its binding seed sequence in *IRF1* are shown in (Fig. 5C). As shown in (Fig. 5D), luciferase activity in the DF-1 cell lines significantly (P = 0.0002) decreased when a miR-23b mimic was co-transfected with pmir-GLO-IRF1-WT containing a miR-23b binding sequence, whereas luciferase activity did not significantly (P = 0.0325) change when transfected with pmir-GLO-IRF1-MT, indicating that miR-23b may directly target the chicken *IRF1* 3′ UTR.

### **In vitro** experiments show that *IRF1* and miR-23b could affect ALV-J replication

The genome of ALV-J is composed of three coding genes: *gag*, *pol* and *env*. The *gp85* protein, a viral envelope polypeptide coded by the *env* gene, is the determinant of subgroup specificity, neutralization and receptor binding^28^. To study the function of *IRF1* and its regulator miR-23b, which is involved in ALV-J invasion, transfected HD11 cells were challenged with ALV-J, and then qPCR and Western blot were used to detect the expression levels of *gp85*.

**Figure 6:**
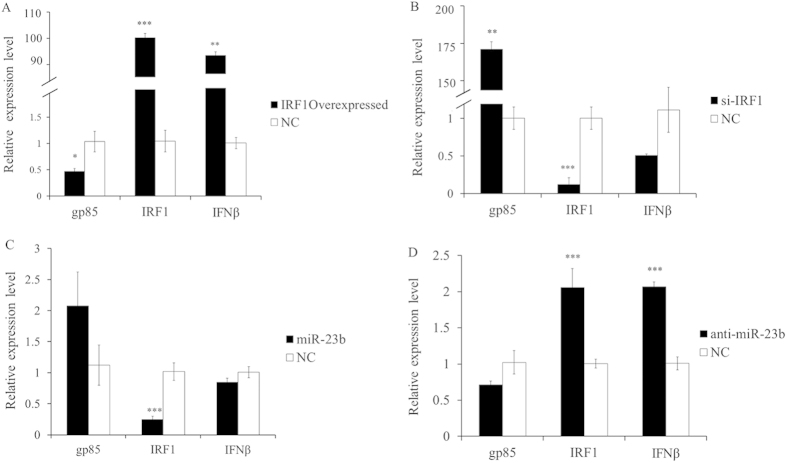
Quantitative analysis of the effects of IRF1 and miR-23b on ALV-J replication. Total RNAs were isolated at 72 h after infection for real-time PCR analysis of the expression of viral *gp85* gene. NC indicated negative control. (**A**) HD11 cells were transfected with the *IRF1* overexpression plasmid, and its inhibitory effects against ALV-J were detected by qPCR. (**B**) HD11 cells transfected with si-IRF1. (**C**) HD11 cells transfected with miR-23b mimic. (**D**) HD11 cells transfected with anti-miR-23b. Data are presented as means ± S.E.M. of three independent experiments. *, **, and *** indicate P-value significance at the threshold levels of 0.05, 0.01 and 0.001, respectively.

**Figure 7:**
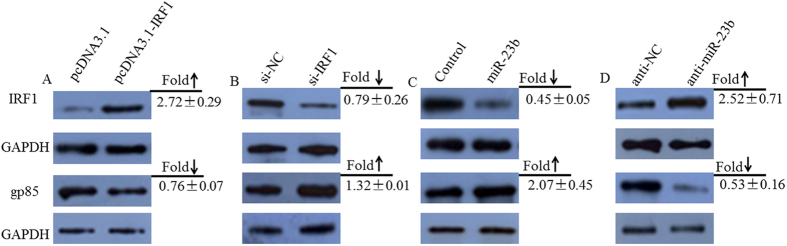
Western Blot analysis of the effects of IRF1 and miR-23b on ALV-J replication. Total proteins were isolated at 72 h after infection for Western blot analysis of the expression of *IRF1* and viral *gp85* gene. (**A**) HD11 cells were transfected with the *IRF1* overexpression plasmid, and its inhibitory effects against ALV-J were detected by Western blot. (**B**) HD11 cells transfected with si-IRF1. (**C**) HD11 cells transfected with miR-23b mimic. (**D**) HD11 cells transfected with anti-miR-23b. Data are presented as means ± S.E.M. of three independent experiments. The full-length blots with IRF1 overexpression, si-IRF1, miR-23b mimic and anti-miR-23b are presented in Supplementary Figure S3 A-H, respectively.

After the cells were transfected with an *IRF1* overexpression plasmid (Fig. 6A), the mRNA expression levels of *IRF1* and *IFNβ* were dramatically up-regulated (P = 0.00002; or P = 0.0014), whereas the expression levels of *gp85* were remarkably decreased ((P = 0.0253). The protein levels of *IRF1* was increased by 2.72-fold, while it was decreased by 0.76-fold for *gp85*, in response to *IRF1* overexpression (Fig. 7A). In contrast, the mRNA expression levels of *gp85* were significantly increased after transfection with si-IRF1 (P = 0.0081), while the expression of *IFNβ* was down-regulated, although this down-regulation was not significant (Fig. 6B). The protein levels of *IRF1* were decreased by 0.79-fold, while it was increased by 1.32-fold for *gp85*, in response to *IRF1* knockdown (si-IRF1) (Fig. 7B). These results indicated that *IRF1* overexpression could inhibit ALV-J replication, whereas its knockdown could enhance ALV-J replication.

The mRNA expression levels of *IRF1* significantly decreased (P = 0.00034) when cells were transfected with a miR-23b mimic (Fig. 6C) and significantly increased (P = 0.00037) after transfection with anti-miR-23b (Fig. 6D), which confirmed that *IRF1* is a target of miR-23b. However, the expression levels of *gp85* increased after transfection with a miR-23b mimic (Fig. 6C) but decreased upon transfection with anti-miR-23b (Fig. 6D), although in both cases, the changes were not statistically significant. miRNA postranscriptionally silence the expression of target genes followed by RNA degradation, but the examples of translational repression without RNA destabilization have also been reported^29^,^30^. This apparent discrepancy is likely due to the fact that miRNAs repress the translation of their targets after initiation with little or no influence on mRNA levels^31^,^32^, and this is consistent with context-dependent miRNA effects^33^,^34^. Actually, the protein levels for *gp85* was either increased by 2.07-fold (Fig. 7C) or was decreased by 0.53-fold (Fig. 7D), in response to miR-23b mimic or anti-miR-23b tranfection. These results suggested that miR-23b overexpression could strengthen ALV-J replication, whereas its loss of function could inhibit ALV-J replication. Moreover, with anti-miR-23b treatment, the expression of *IFNβ* was sharply up-regulated (P = 0.0004).

## Discussion

High-throughput sequencing is a new and powerful technique that enables the mRNA and miRNA transcriptomes in many organisms^35^,^36^,^37^ to be profiled. In the present study, Illumina deep sequencing was performed to investigate the miRNA and mRNA profiles of ALV-J-infected and uninfected chicken spleens. Consequently, a number of differentially expressed miRNAs and genes were identified. After target prediction, miRNAs targets that were also identified as DEGs were selected. Negatively correlated expression patterns between miRNAs and their corresponding targets have been reported^38^. Therefore, in the present study, the interaction network construction was restricted to miRNA-gene pairs that had opposing expression patterns.

Up-regulated miRNAs may act as oncogenes and promote tumor development by targeting and suppressing tumor suppressor genes. miR-23b is highly conserved in most vertebrates and is up-regulated in various human cancers. *IRF1* is inactivated by the deletion of its exon in human acute myelocytic leukemia^21^; however, its inactivation by miRNA in ALV-J-infected chicken has not been reported. In the current study, we focused on miR-23b partly because its potential target gene *IRF1* is known to exert antiviral effects. Through Solexa deep sequencing, we found that miR-23b was up-regulated in ALV-J-infected spleens compared to uninfected spleens, while its target gene *IRF1* had an opposing expression pattern. The qPCR results revealed that the expression patterns of miR-23b and *IRF1* were consistent with the Solexa deep sequencing data. Therefore, we hypothesized that miR-23b might be involved in controlling host immune defenses through the regulation of *IRF1*.

Several previous studies have provided evidence for the involvement of miR-23b in tumorigenesis and viral integration. In human cervical cancer, the oncogenic HPV-16 E6 protein reduces the expression of miR-23b, which subsequently increases the expression of its target *uPA*, and this increased expression may contribute to the migration of human cervical cancer cells^26^. The suppression of miR-23b may reduce cancer cell migration, invasion, growth and angiogenesis in human colon cancer^39^. Acting as an oncogene, miR-23b expression is up-regulated in glioma, and miR-23b down-regulation could suppress glioma cell growth and invasion by targeting the *VHL* gene^40^. During virus invasion, miR-23b exhibits antiviral functions against rhinoviruses by down-regulating its target *VLDLR*, which acts as a receptor for rhinovirus infection^27^. Similarly, this study found that miR-23b was up-regulated in ALV-J-infected chicken spleens. The expression of ALV-J *gp85* was remarkably increased in challenged HD11 cells that were transfected with a miR-23b mimic. These results suggested that up-regulated miR-23b may be associated with ALV-J invasion and avian leukosis formation.

A previous study demonstrated that *IRF1* is a target of miR-23a in human gastric adenocarcinoma cells^25^. In the current study, *IRF1* was predicted to be a potential miR-23b target by the miRNA-target interaction network, and opposing expression patterns for miR-23b and *IRF1* were identified. Furthermore, the role of miR-23b as a regulatory factor of *IRF1* was confirmed by the dual-luciferase reporter assay.

Interferon regulatory factors *(IRFs*), which possess a unique “tryptophan cluster” domain for binding to Type I interferon (*IFNα* and *IFNβ*), are a family of transcription mediators that are involved in the regulation of interferon expression^41^. The importance of *IRFs* is demonstrated by their role in antiviral defense, the immune response and hematopoietic differentiation^42^. Vertebrates express ten *IRFs* (denoted 1-10) that possess considerably different functions and catalytic activities. *IRF1*, which was the first identified *IRF*, acts as a critical regulatory protein of the inflammatory response and functions as a tumor suppressor that is involved in cell cycle progression and apoptosis^43^. The down-regulation of *IRF1* expression by miRNAs has been reported in a variety of human cancers. For example, miR-23a induces gastric adenocarcinoma formation through silencing *IRF1* expression^25^. Conversely, *IRF1* overexpression in cancer cell lines suppresses cancer cell cycle transition, apoptosis and caspase activation^44^. Moreover, *IRF1* possesses certain substrates including *IFNβ*, which is a key gene of Toll-like reporter signaling pathways, and exerts antivirus activity^22^. *IRF1* can bind to the promotor of *IFNβ* and activate its expression^41^. In this study, the expression levels of *gp85* were significantly down-regulated after transfection with an *IRF1* overexpression plasmid. Importantly, *IRF1* and *IFNβ* mRNA levels sharply increased. When the expression of *IRF1* was silenced with si-IRF1, *gp85* levels increased and *IFNβ* mRNA levels decreased. These results indicated that *IRF1* could inhibit ALV-J replication by activating *IFNβ* expression.

In summary, our study presented evidence that the expression of miR-23b was up-regulated in ALV-J-infected chicken spleens and that *IRF1* is a target of miR-23b. During ALV-J invasion, miR-23b up-regulation could decrease the expression of *IRF1* and further down-regulate the expression of *IFNβ*. This finding provides novel insights into the involvement of miR-23b in ALV-J infection.

## Methods

### Ethics statement

The animal experiments were approved by the South China Agricultural University Institutional Animal Care and Use Committee (approval ID: SCAU#0011). And the experiment was performed in accordance with the regulations and guidelines established by this committee.

### Sample selection and virus isolation

The clinical symptoms of 140-day-old spontaneous infection female White Recessive Rock (WRR) chickens were as follows: depression and hemorrhages in the skin of the phalanges and feather follicles. In total, 23 tumor spleen samples and 23 healthy spleen samples from chickens were collected independently. The procedures for virus isolation were performed in DF-1 cells as described previously^45^. After the infected DF-l cells were cultured for 7 days, the supernatants of these cells were harvested and tested for ALV group-specific antigen (p27) using an antigen-capture ELISA kit (IDEXX Laboratories, USA). PCR was used to test genomic DNA from cultured DF-1 cells. Previously published specific primers (Supplementary Table S5) were synthesized and employed to detect different subgroups of exogenous ALVs^46^ and other avian tumor viruses, including MDV^47^ and REV^48^. ALV-J infection was further confirmed by IFA using standard techniques^49^. The images of infected cells were collected by fluorescence microscope (Nikon, Japan) using NIS-Elements BR analysis software (Nikon). Finally, spleens from the two groups (ALV-J-infected group: WRR^+^; uninfected group: WRR^−^) were subjected to Illumina deep sequencing.

### Total RNA isolation, cDNA library construction and Illumina deep sequencing

Total RNA from each of three ALV-J-infected spleens (designated WRR_1_^+^, WRR_2_^+^, WRR_3_^+^) and three uninfected normal spleen samples (designated WRR_1_^−^, WRR_2_^−^, WRR_3_^−^) was isolated using TRIzol reagent following the manufacturer’s instructions (Invitrogen). Then, RNA samples from three individuals within each group were pooled in equal amounts.

For RNA-seq, two cDNA libraries (infected and uninfected groups) were prepared using a Truseq^TM^ RNA sample prep kit (Illumina) according to the manufacturer’s protocol. The protocol consisted of the following processes: mRNA containing poly (A) segments was purified from 10 μg of total RNA using oligo (dT) magnetic beads, chemically fragmented into 200-500 bp fragments, and then reverse transcribed into cDNA. Thereafter, Illumina adapters were ligated to the cDNA fragments, and the two samples were sequenced on a Genome Analyzer IIx (Illumina).

For small RNA-seq, fragments with lengths of 16-30 nt were obtained from total RNA by denaturing 15% PAGE. Subsequently, these small RNAs were ligated with 5′ and 3′ RNA adapters, followed by reverse transcription and amplification with 15 PCR cycles to create cDNA constructs. The amplified cDNA constructs were quantified using an Agilent 2100 bioanalyzer and a DNA 1000 Nano Chip Kit (Agilent). Small RNA sequencing was performed on an Illumina HiSeq 2500 platform (Illumina)

### RNA-seq read processing, annotation and differential expression analysis

After RNA-seq, the raw reads were first filtered by removing low-quality reads (Q value < 25) and adapter sequences using FASTX-Toolkit software ( http://hannonlab.cshl.edu/fastx_toolkit/). The filtered reads were mapped to the chicken reference genome using TopHat. The clean reads of two samples (WRR^+^and WRR^−^) were assembled and conjoined into contigs using Trinity ( http://trinityrnaseq.sourceforge.net/). The resulting contigs from the same transcript were recognized by overlapping sequences and further connected into unigenes. These unigenes were further annotated by BLAST searching against the Nr (NCBI non-redundant protein sequences), GO and KEGG databases. To estimate the expression of each gene, the total number of reads mapped to its unigenes were calculated and normalized to FPKM (fragments per kilobase of transcript per million mapped reads). DEGs were identified with the R package DEGseq using a Benjamini q-value of 0.05 (cut-off at 5% false discovery rate [FDR]). GO and KEGG functional enrichment analyses were performed with the GOseq package using a corrected P-value of less than 0.05 as the threshold.

For small RNA sequencing, the generated raw reads were processed with in-house Perl scripts to filter low quality reads and adaptor dimers. The remaining clean reads were used to calculate the length distribution with FASTX-Toolkit software ( http://hannonlab.cshl.edu/fastx_toolkit/). All identical clean reads were counted and assembled into unique sequences. The resulting unique sequences were matched with the Rfam database 10.1 ( http://rfam.sanger.ac.uk/) to eliminate rRNA, tRNA, snRNA and other ncRNA sequences. Subsequently, the remaining unique sequences were blasted against the chicken precursors in miRBase 18.0 ( http://www.mirbase.org/) to screen out candidate miRNAs using the miRDeep2 program ( http://www.mdc-berlin.de/en/research/research_teams/systems_biology_of_gene_regulatory_elements/projects/miRDeep/). miRNA differential expression was based on normalized clean read counts that were identified by the R package DEGseq, using a Benjamini q-value of 0.001 as the cut-off.

The sequencing data obtained from RNA-Seq and small RNA-Seq were released to the GEO database under the accession numbers GSE63226 and GSE63676, respectively.

### The network construction of differentially expressed miRNAs and genes

To identify the key genes or miRNAs involved in the response against ALV-J infection, we integrally analyzed the differential expression mRNA-miRNA pairs and constructed their interaction networks. Network construction involved the following two components: interactions between miRNAs and targets and interactions between targets and targets. First, the putative target genes of differentially expressed miRNAs were predicted with miRDB^50^ and TargetScan^18^. Previous studies have reported an inverse correlation between the expression patterns of miRNAs and their targets^42^. Therefore, those targets whose mRNA expression patterns were in opposition to their corresponding miRNAs were selected as candidate targets for differentially expressed miRNAs. Additionally, STRING, a database of known and predicted protein interactions, was used to construct target-target interactions. To determine the key gene involved in the host anti-ALV-J response, GO enrichment annotation of target genes that were selected to construct the interaction network was also performed with STRING. Finally, miRNA-target and target-target interactions were integrated to construct a possible regulatory network using Cytoscape.

### RT-PCR and quantitative real-time PCR (qPCR)

Total RNA was extracted from frozen tissues or cell lines using RNAiso reagent (TaKaRa) according to the manufacturer’s protocol. For mRNA expression analysis, cDNA synthesis of mRNA was performed using a PrimeScript RT Reagent Kit (Perfect Real Time) (TaKaRa). The qPCR primers were designed by Primer Premier 5.0 (Supplementary Table S6). The *β-actin* gene was used as an internal control. For miRNA expression analysis, reverse transcription was performed using a ReverTra Ace qPCR RT Kit (Toyobo) with a gga-miR-23b bulge-loop RT primer. The bulge-loop RT primer and qPCR primers specific for gga-miR-23b were designed and synthesized by RiboBio (RiboBio). The *U6* gene was used as an internal control. qPCR reactions were performed on a Bio-Rad CFX96 Real-Time Detection System using an iTaq^TM^ Universal SYBR^®^ Green Supermix Kit (Bio-Rad). Data analyses were performed using the 2^−ΔΔCt^ method^39^.

### RNA oligoribonucleotides and plasmids

The miR-23b mimics, mimic control duplexes, anti-miR-23b antagomirs, anti-NC, siRNA target against the *IRF1* gene (si-IRF1) and siRNA nonspecific control duplex were designed and synthesized by GenePharma (GenePharma). To construct a miR-23b target luciferase reporter (pmir-GLO-IRF1-WT), the segment sequence of the *IRF1* 3′ UTR (616 bp) that contained the putative miR-23b binding sequence was amplified by PCR using a cDNA template synthesized from total RNA. Then, the PCR product was subcloned into XhoI*/*SalI restriction sites in the pmirGLO dual-luciferase reporter vector (Promega) to generate the pmir-GLO-IRF1-WT reporter. To generate a miR-23b target-mutated reporter (pmir-GLO-IRF1-Mut), mutations were achieved by changing the miR-23b binding seed sequences from AATGTGA to CCGAGTG using the megaprimer PCR method. The *IRF1* overexpression construct was generated by amplifying the *IRF1* coding sequence, which was subsequently integrated into the NheI*/*XhoI restriction sites of the pcDNA3.1 overexpression plasmid (Promega). The construct was designated pcDNA3.1-IRF1.

### Luciferase reporter assay

DF-1 cells were seeded in 96-well plates one day before transfection and then co-transfected with 100 ng of *IRF1* 3′ UTR wild-type or mutant constructs and 100 nM of miR-23b mimics or mimic control duplexes using Lipofectamine 2000 reagent. After 48 h, luciferase activity analysis was performed using a Fluorescence/Multi-Detection Microplate Reader (BioTek) and a Dual-GLO^®^ Luciferase Assay System Kit (Promega).

### Transfection of pcDNA3.1-IRF1, si-IRF1, miR-23b mimics, and anti-miR-23b and preparation of ALV-J

Before transfection, HD11 cells were seeded in 12-well plates. When the cells grew to 80% confluence, they were transfected with (I) 1.6 μg of pcDNA3.1-IRF1; (II) 100 nM si-IRF1; (III) 100 nM miR-23b mimic and (IV) 100 nM anti-miR-23b using Lipofectamine 2000 reagent. After incubation for 4 h, Lipofectamine 2000 transfection reagent was removed, and the cells were replenished with DMEM supplemented with 10% fetal calf serum. Twelve hours later, the transfected cells were inoculated with 50% tissue culture infectious doses (TICD_50_) of ALV-J. After incubation for 2 h, the virus-containing supernatants were discarded, and the infected cells were replenished with DMEM medium containing 1% fetal calf serum. After the infected cells were incubated for 72 h, the infection level were analyzed by qPCR.

### Western Blot

The primary antibodies were as follows: rabbit polyclonal anti-IRF1 (LSBio, USA), ALV-J-specific monoclonal antibody JE-9 kindly provided by Prof. Aijian Qin (Yangzhou University, China) and goat polyclonal anti-GAPDH (LSBio). Rabbit polyclonal (IgG) to chicken IgG (LSBio) serve as secondary antibody. After transfection with pcDNA3.1-IRF1, si-IRF1, miR-23b mimic and anti-miR-23b, HD11 cells were inoculated with 50% tissue culture infectious doses (TICD_50_) of ALV-J. 72 h later, HD11 cells were subjected to Western blot analysis as previously report^24^.

## Author Contributions

Conceived and designed the experiments: X.Z., Q.N. Performed the experiments: Z.L. Analyzed the data: B.C., M.F., Y.O., M.Z., Q.Y., H.X. Contributed materials: Q.N. Wrote the paper: Z.L.

## Additional Information

**How to cite this article**: Li, Z. *et al.* MircoRNA-23b Promotes Avian Leukosis Virus Subgroup J (ALV-J) Replication by Targeting *IRF1*. *Sci. Rep.* **5**, 10294; doi: 10.1038/srep10294 (2015).

## Supplementary Material

Supplementary Information

## Figures and Tables

**Table 1:** Target genes in the interaction network that involved host immune process.

**Target gene**	**Description**	**Regulated miRNAs**
IRF1	Interferon regulatory factor 1	miR-23b; miR-2964
MX1	Myxovirus (influenza virus) resistance 1, interferon-inducible protein p78 (mouse)	miR-460b-5p
NMI	N-myc (and STAT) interactor	miR-15a; miR-383
IFIH1	Interferon induced with helicase C domain 1	miR-34b; miR-2188
