# Supplementary material for: MicroRNA-23b Promotes Avian Leukosis Virus Subgroup J (ALV-J) Replication by Targeting IRF1
Source: Sci Rep. 2015 May 18;5:10294. doi: 10.1038/srep10294 (PMC4434839; doi:10.1038/srep10294)
Supplement: Supplementary Information [file srep10294-s1.pdf]

MicroRNA-23b Promotes Avian Leukosis Virus Subgroup J (ALV-J) Replication by Targeting *IRF1*

Zhenhui Li[1, 2], Biao Chen[1, 2], Min Feng[1, 2], Hongjia Ouyang[1, 2], Ming Zheng[1, 2], Qiao Ye[1, 2], Qinghua Nie[1, 2, *], and Xiquan Zhang[1, 2]

[1]Department of Animal Genetics, Breeding and Reproduction, College of Animal Science, South China Agricultural University, Guangzhou 510642, Guangdong, China.

[2]Guangdong Provincial Key Lab of Agro-Animal Genomics and Molecular Breeding and Key Lab of Chicken Genetics, Breeding and Reproduction, Ministry of Agriculture, Guangzhou 510642, Guangdong, China.

*To whom correspondence should be addressed. Tel: 86-20-85285759; Fax: 86-20-85280740; Email: nqinghua@scau.edu.cn.

Outline of Supplementary Information

Supplementary Figures.

Figure S1. Gene ontology enrichment analysis.

Figure S2. PCR detection for ALV-J, exogenous ALVs (A-D), MDV and REV.

Figure S3. Full-length blots.

Supplementary Tables.

Table S1. Differential expression analysis of miRNAs in ALV-J infected compared with uninfected chicken spleens using DEG.

Table S2. Differential expression analysis of genes in ALV-J infected compared with uninfected chicken spleens using DEG.

Table S3. Differentially expressed miRNAs and theirs corresponding target genes with differential expression in ALV-J infected compared with uninfected chicken spleens.

Table S4. Gene ontology (GO) function annotation of the 111 genes was also performed by STRING.

Table S5. Primers pairs employed to indentify ALVs, MDV and REV.

Table S6. Real-time PCR primers for IRF1, gp85, IFNβ and β-active.

Supplementary Figures

Figure S1 Gene ontology enrichment analysis.

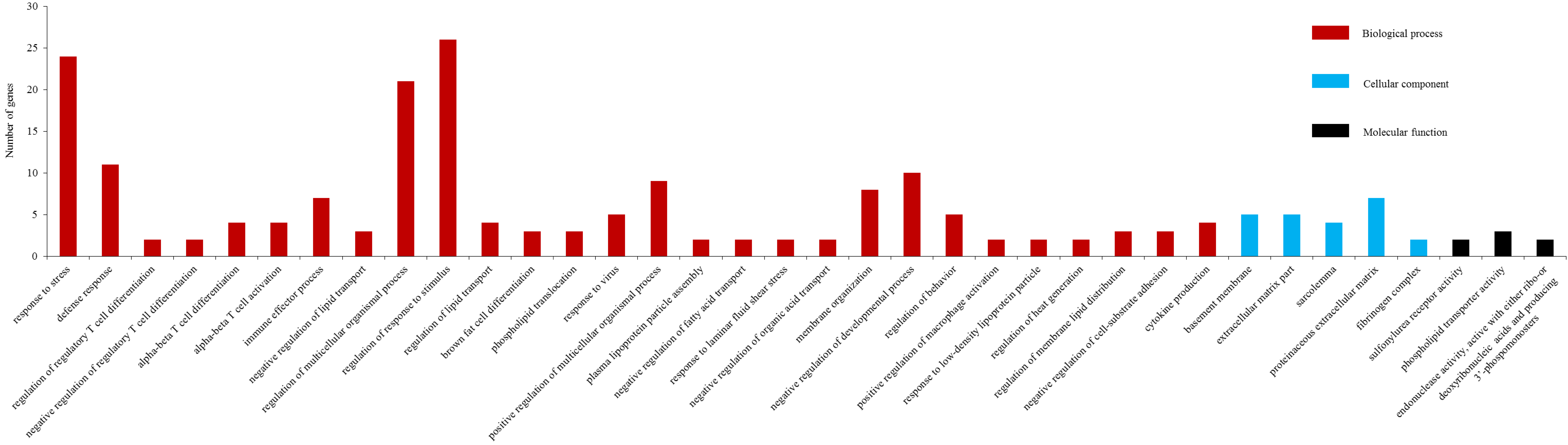

Figure S2 A: ALV-J was detected by PCR

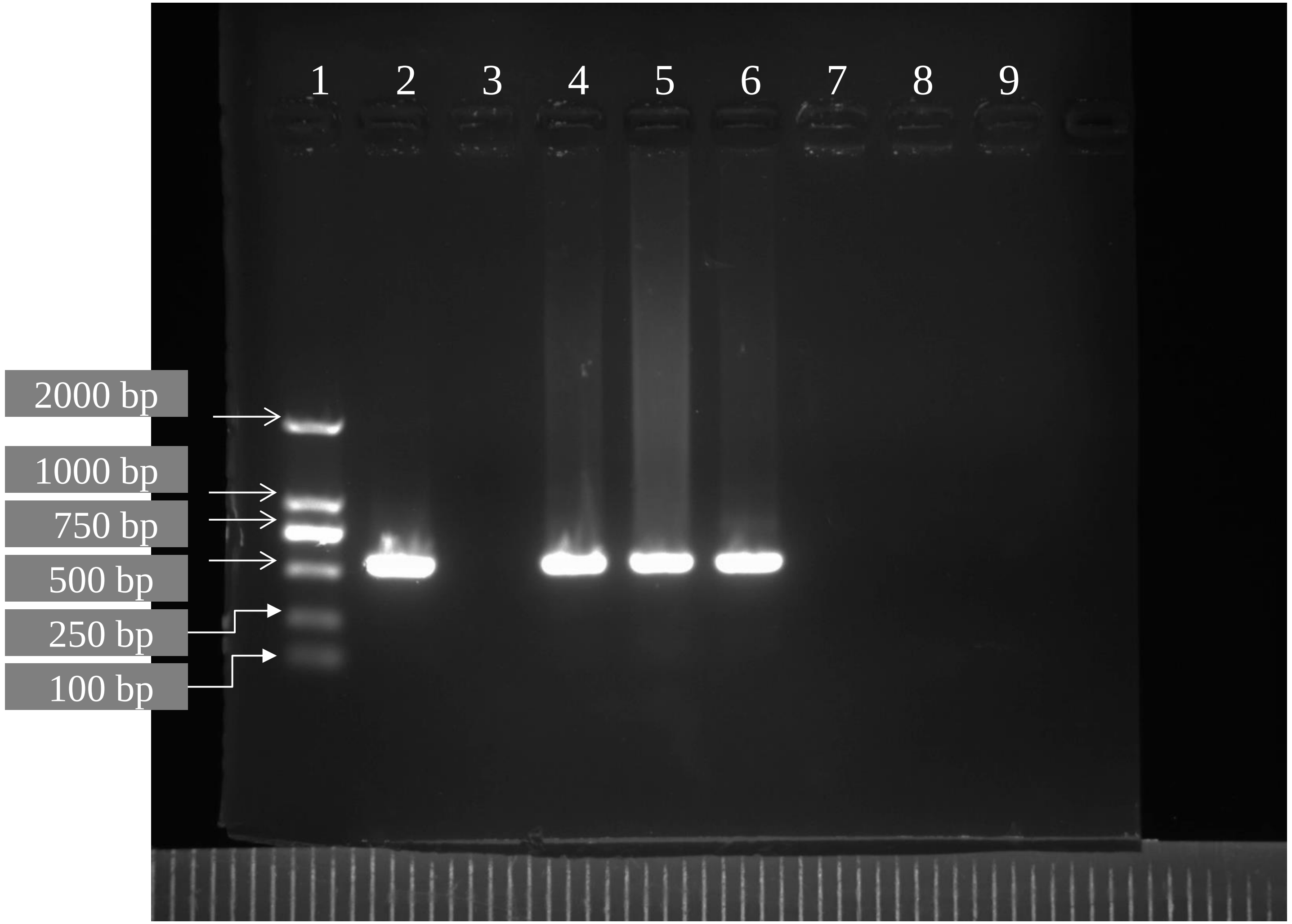

Lane	
1	DL2000 Maker
2	Positive control
3	Negative control
4	WRR_1^+
5	WRR_2^+
6	WRR_3^+
7	WRR_1^-
8	WRR_2^-
9	WRR_3^-

Figure S2 B: Exogenous ALVs (A-D) was detected by PCR

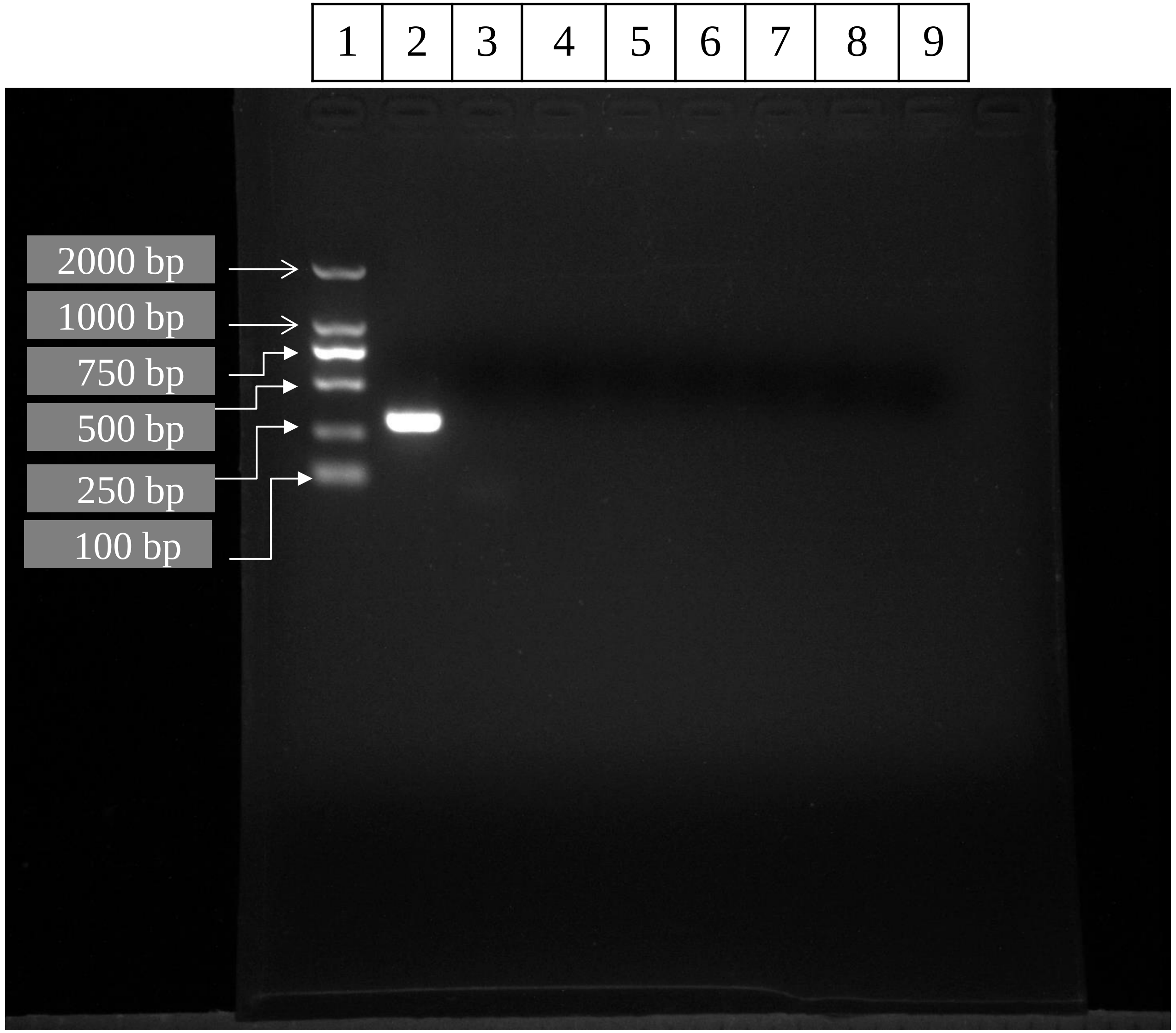

Lane	
1	DL2000 Maker
2	Positive control
3	Negative control
4	WRR_1^+
5	WRR_2^+
6	WRR_3^+
7	WRR_1^-
8	WRR_2^-
9	WRR_3^-

Figure S2 C: MDV was detected by PCR

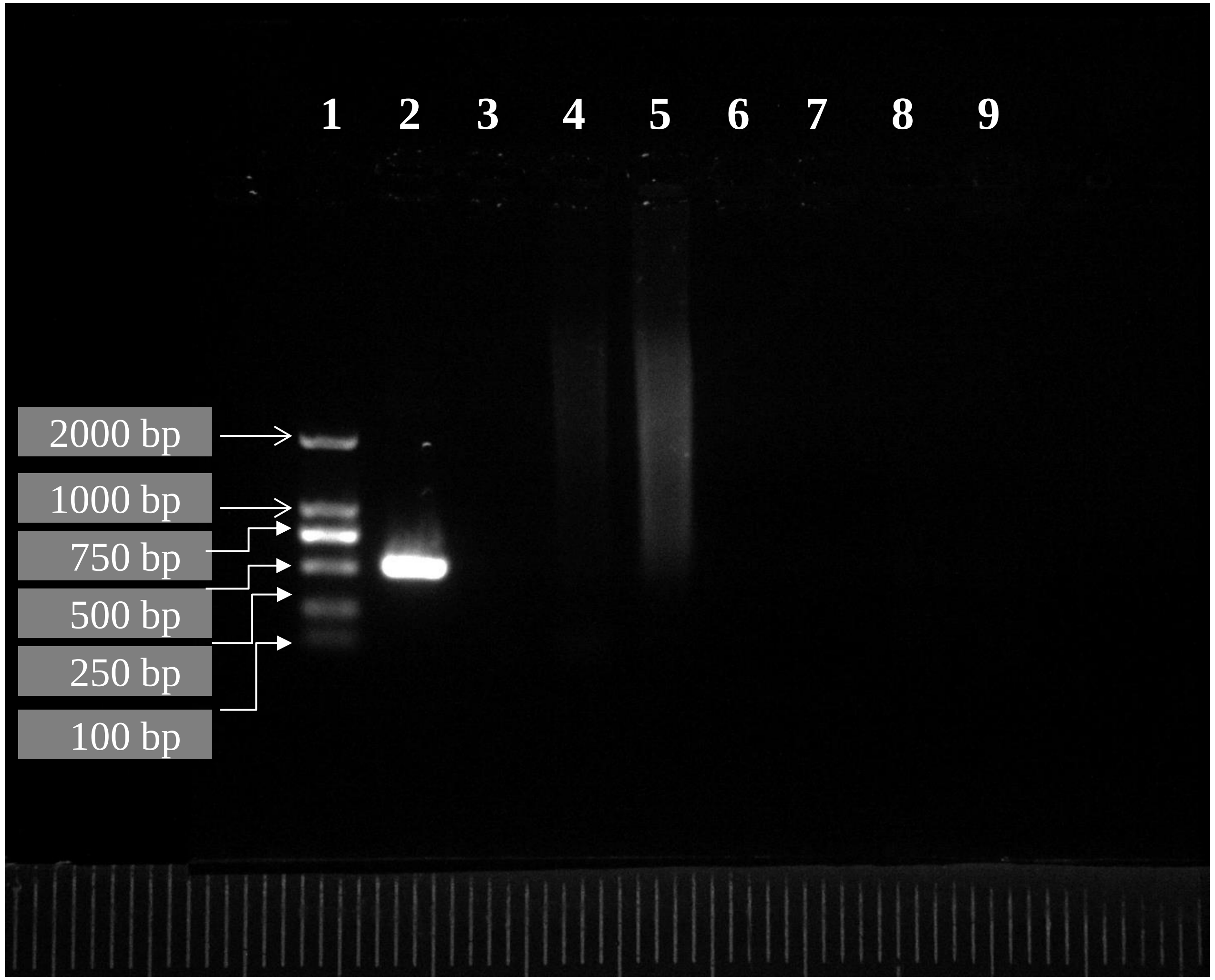

Lane	
1	DL2000 Maker
2	Positive control
3	Negative control
4	WRR_1^+
5	WRR_2^+
6	WRR_3^+
7	WRR_1^-
8	WRR_2^-
9	WRR_3^-

Figure S2 D: REV was detected by PCR

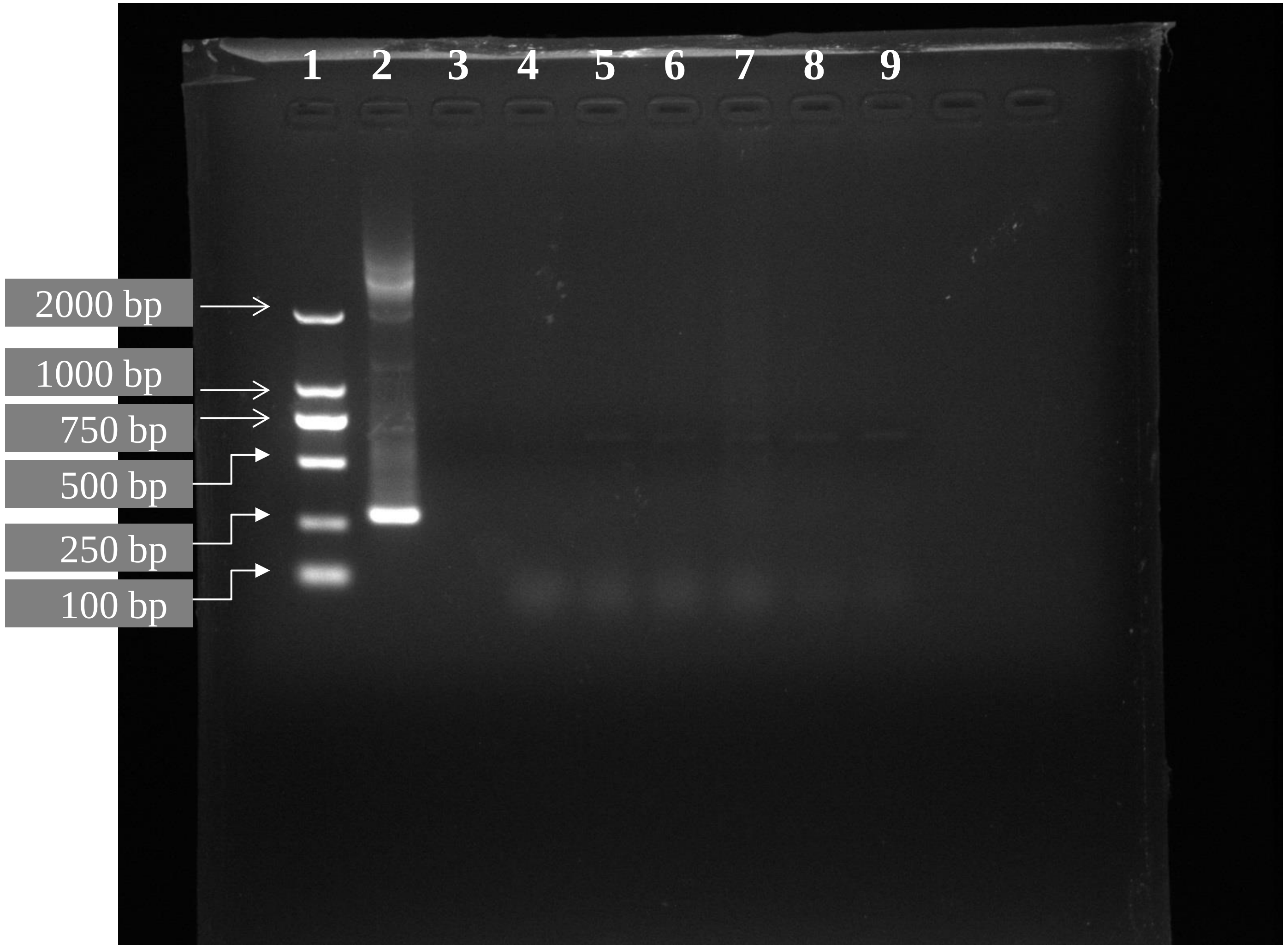

Lane	
1	DL2000 Maker
2	Positive control
3	Negative control
4	WRR_1^+
5	WRR_2^+
6	WRR_3^+
7	WRR_1^-
8	WRR_2^-
9	WRR_3^-

Figure S3 A (For IRF1)

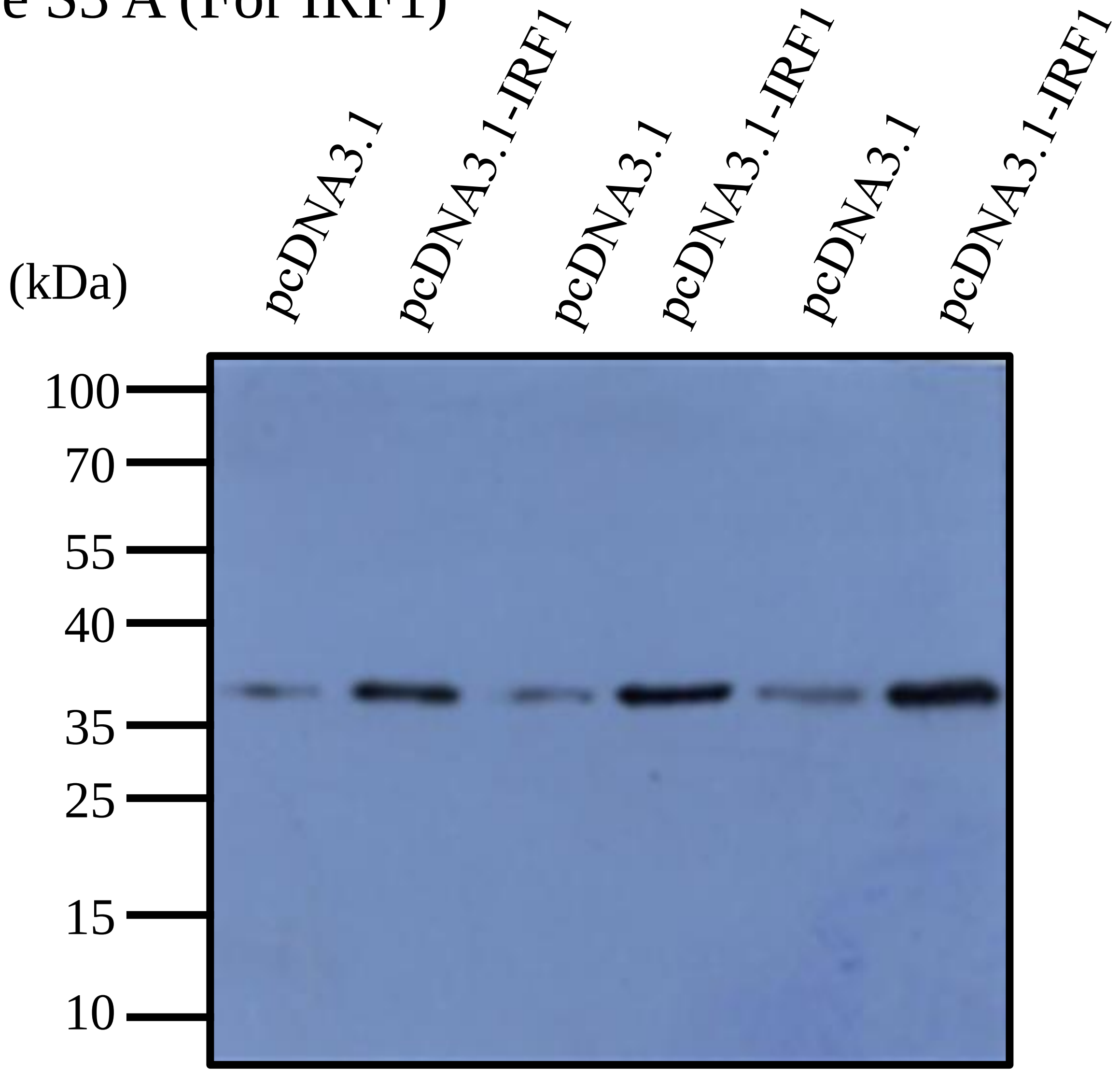

Figure S3 B (For IRF1)

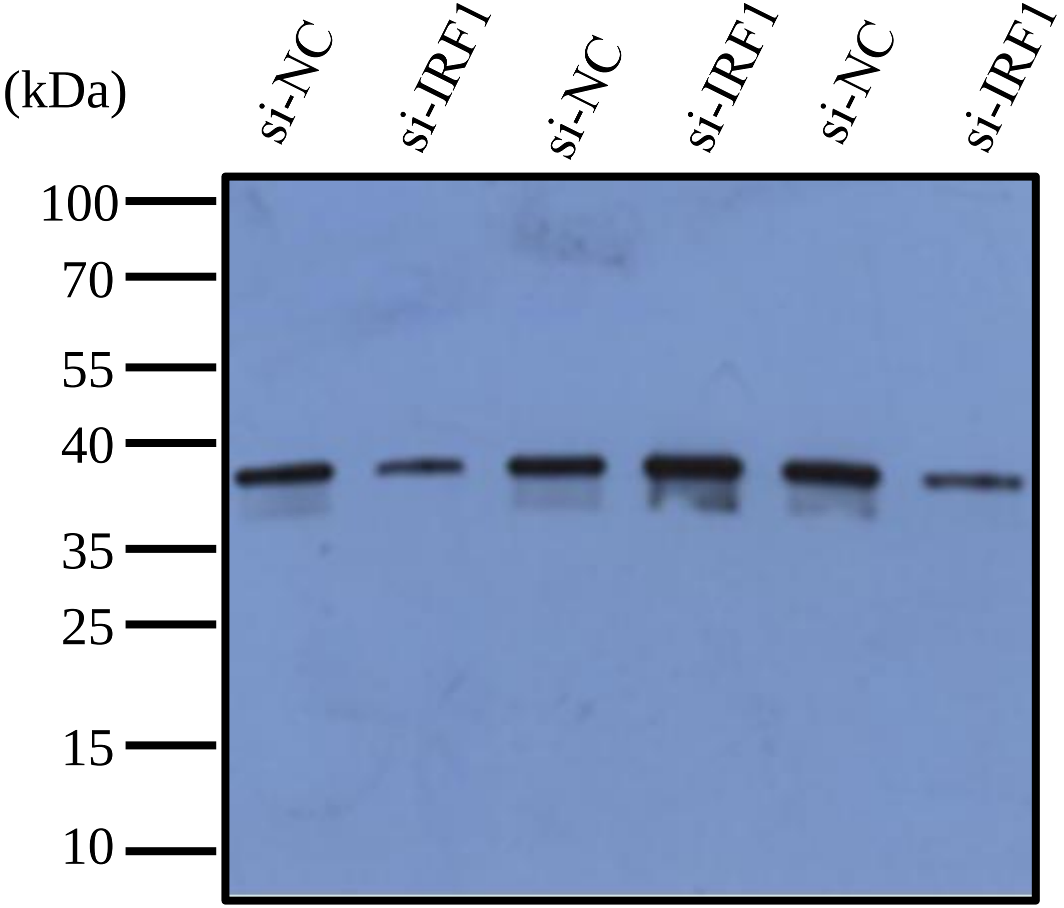

Figure S3 C (For IRF1)

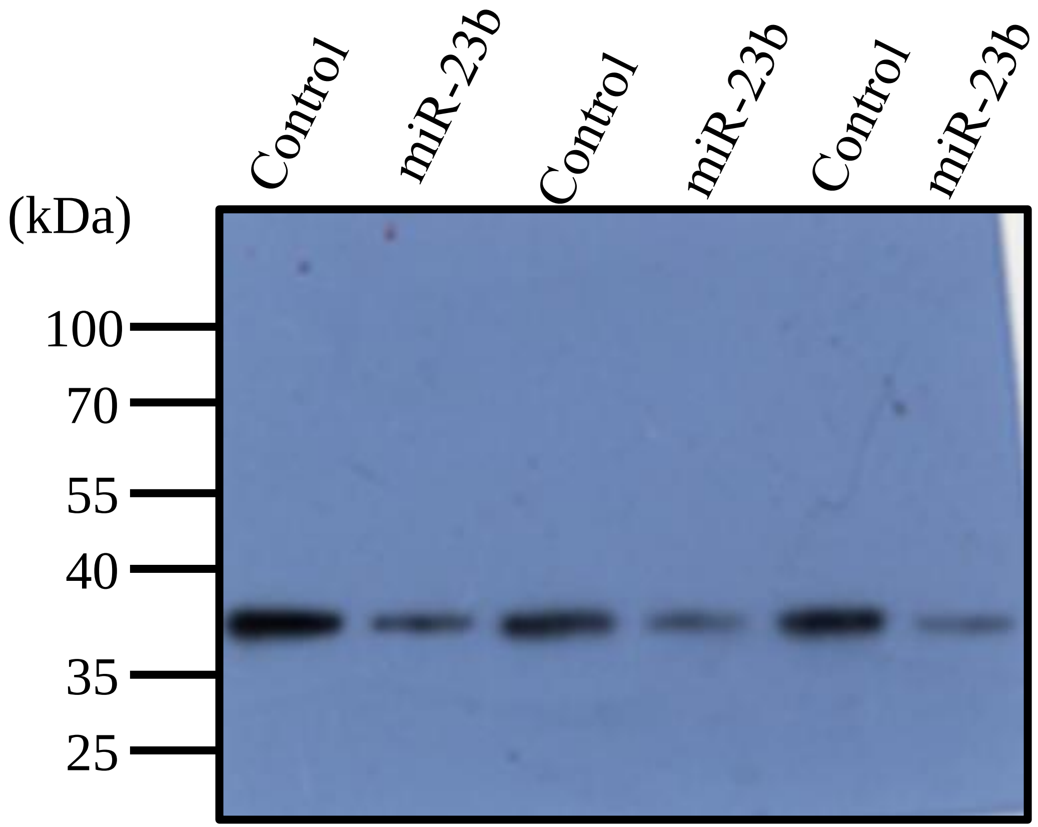

Figure S3 D (For IRF1)

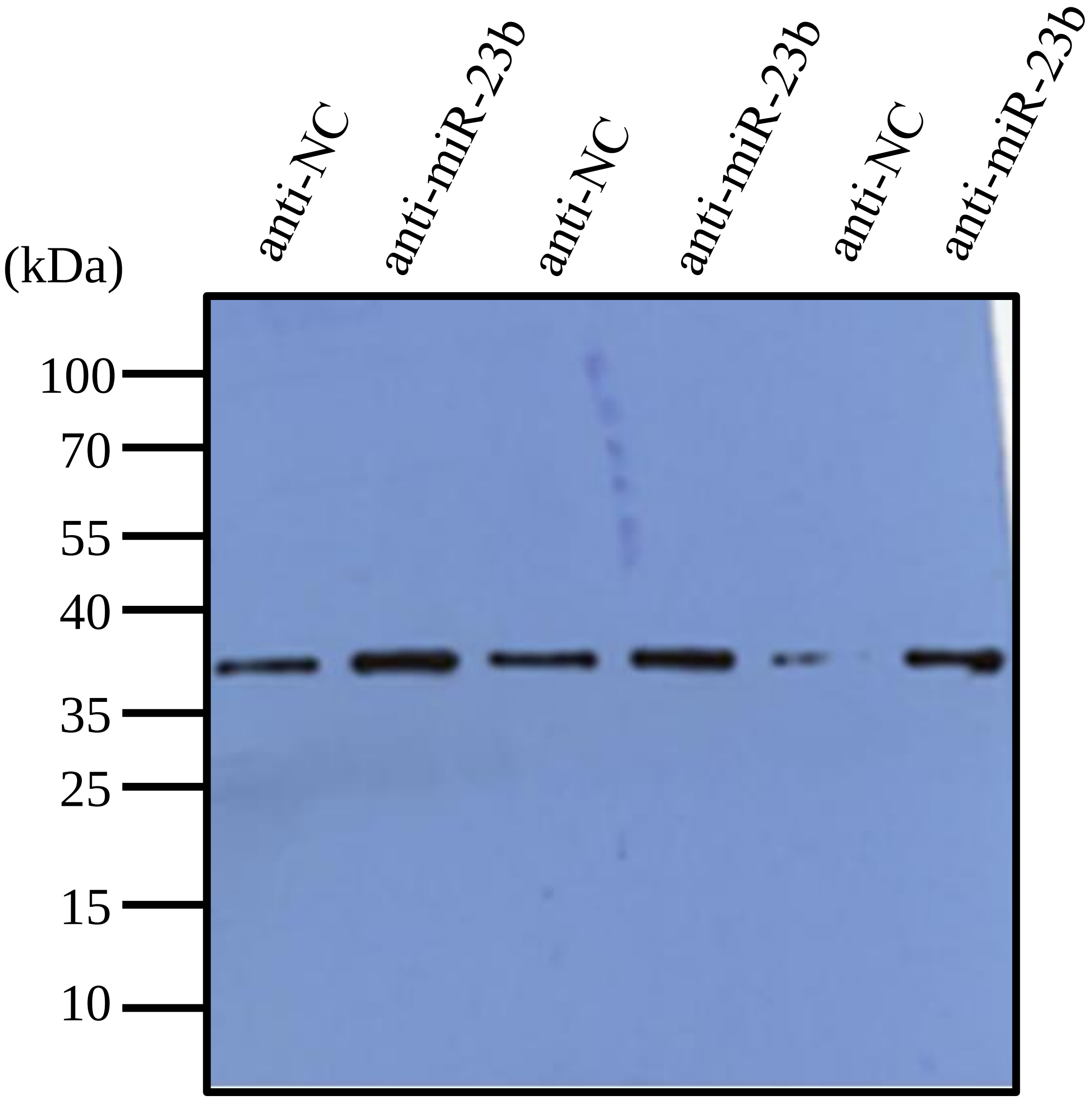

Figure S3 E (For gp85)

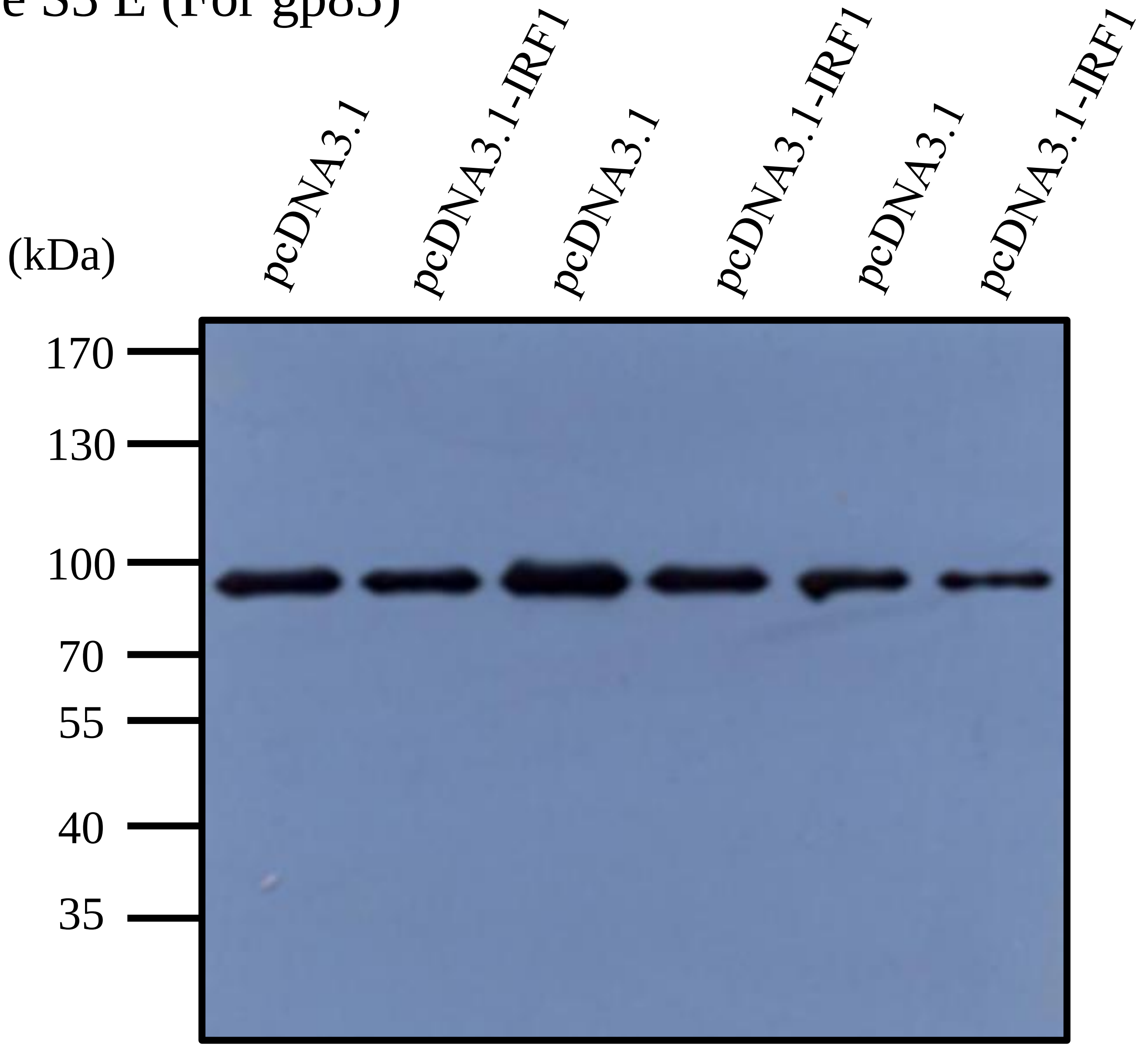

Figure S3 F (For gp85)

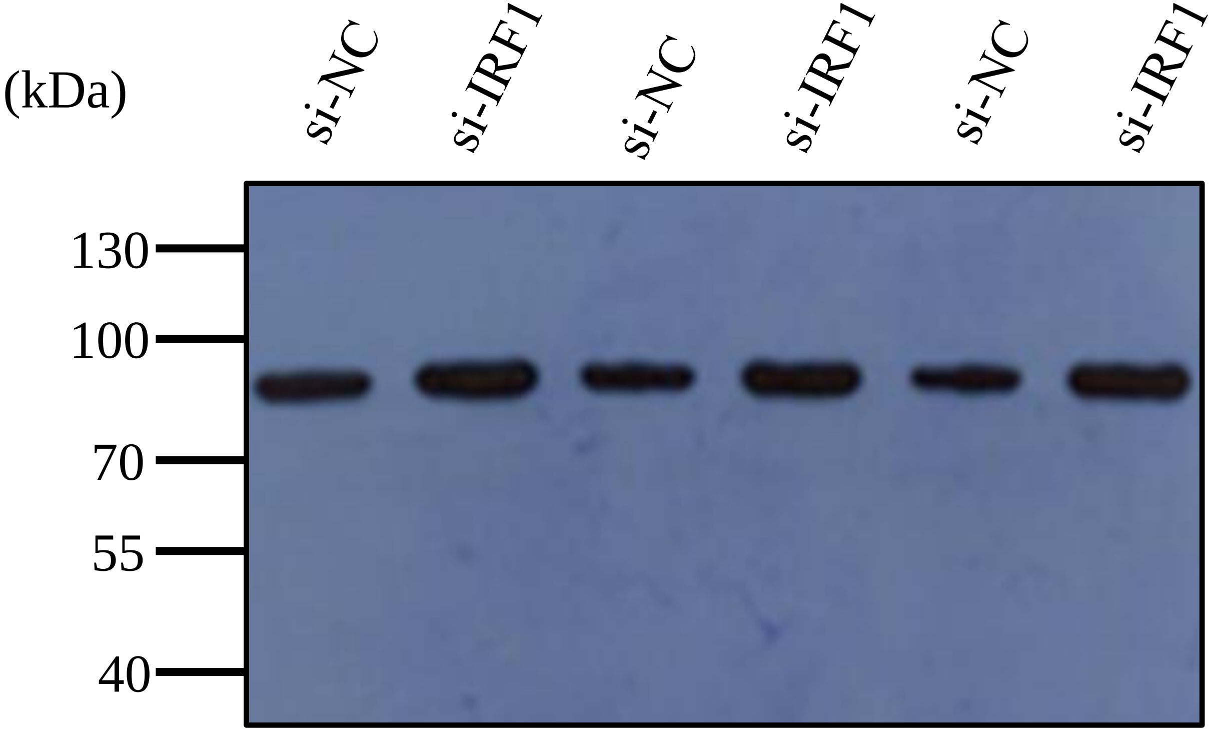

Figure S3 G (For gp85)

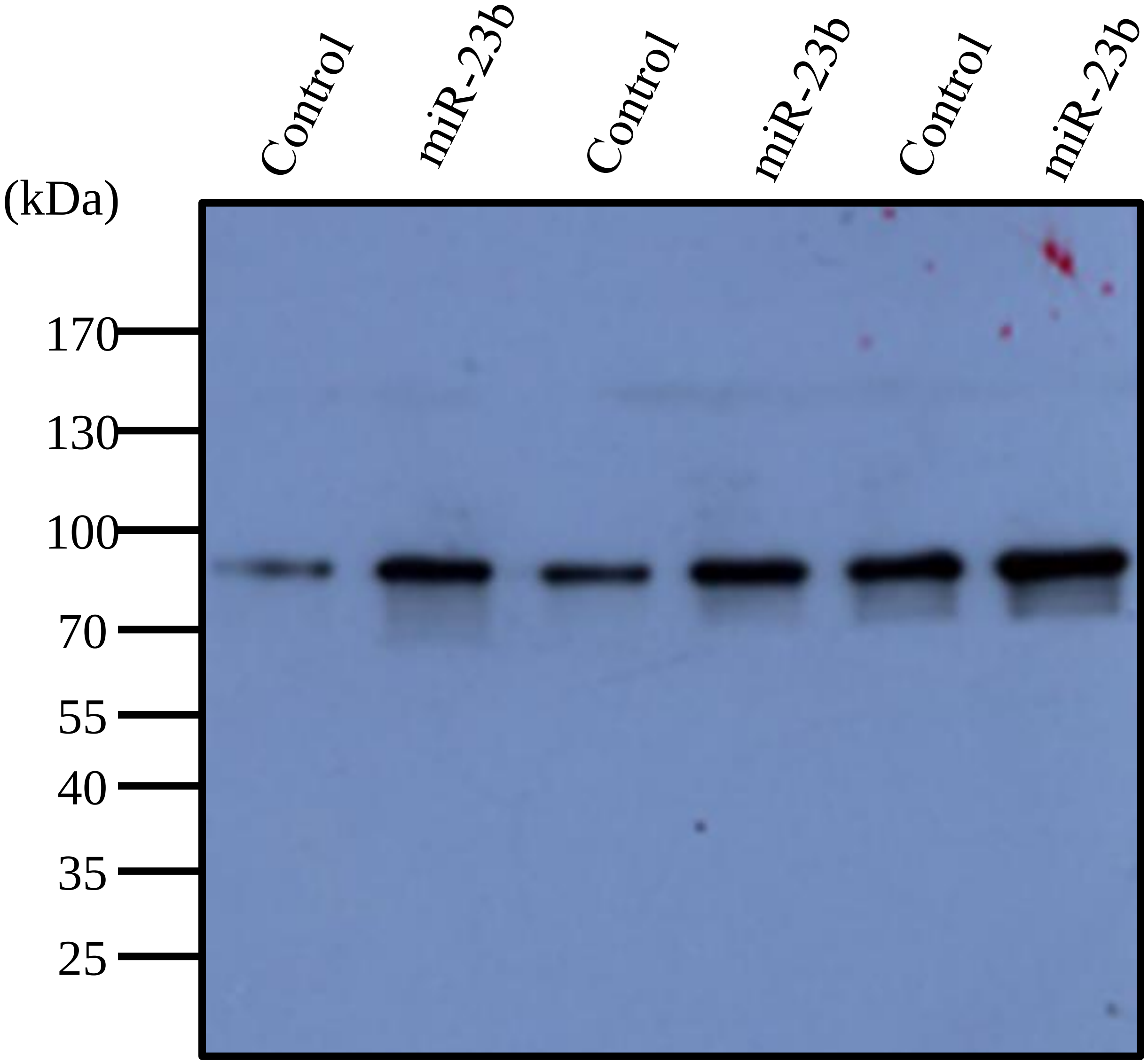

Figure S3 H (For gp85)

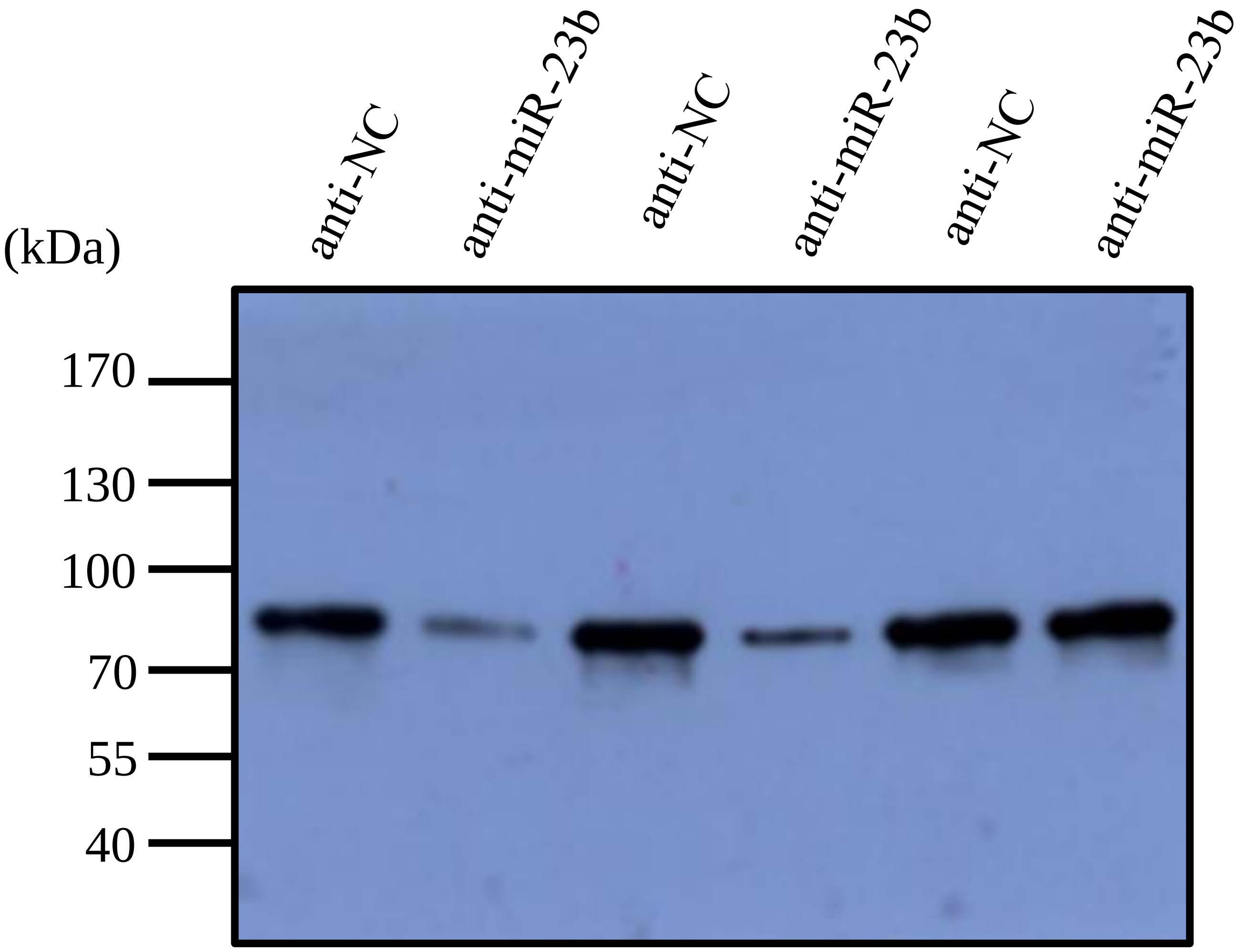

Supplementary Tables.

Table S1. Differential expression analysis of miRNAs in AVL-J infected compared with uninfected chicken spleens using DEG.

miRNA name	ALV-J infected spleens (WRR^+) FPKM	Uninfected spleens (WRR^-) FPKM	log2 Ratio(WRR^+/WRR^-)	q value	Regualtion[a]
gga-miR-10a-5p	11492986.7200	10095802.8900	0.1870	0	up
gga-miR-10b	9358782.6500	8271177.7400	0.1782	0	up
gga-let-7c	1206765.5800	888120.1100	0.4423	0	up
gga-let-7j	2472908.1900	2066848.0700	0.2588	0	up
gga-let-7f	2489453.1800	2100290.0400	0.2452	0	up
gga-miR-181a-5p	3651195.6500	3273091.2900	0.1577	0	up
gga-miR-30c	419880.0200	323629.2100	0.3756	0	up
gga-miR-26a	6688359.3900	6171874.2100	0.1159	0	up
gga-let-7b	219225.5900	155761.7100	0.4931	0	up
gga-let-7k	1423599.8300	1253924.5900	0.1831	0	up
gga-let-7g	415491.9700	344797.1400	0.2691	0	up
gga-miR-126-5p	734929.9200	649284.9800	0.1788	0	up
gga-miR-92	1955472.2000	1805797.3200	0.1149	0	up
gga-miR-126-3p	251561.6500	212306.3900	0.2448	0	up
gga-miR-30b	89639.8200	73254.1900	0.2912	2.08E-267	up
gga-miR-181b	154771.3800	132460.1600	0.2246	5.52E-255	up
gga-miR-125b	342385.3600	306407.3300	0.1602	1.77E-244	up
gga-miR-204	18812.6400	12564.6400	0.5823	7.28E-237	up
gga-miR-211	18812.6400	12564.6400	0.5823	7.28E-237	up
gga-miR-30d	2395549.0000	2262888.5000	0.0822	1.05E-219	up
gga-let-7i	282160.1700	252057.4400	0.1628	3.88E-210	up
gga-miR-223	114460.2600	97374.0100	0.2332	2.84E-206	up
gga-miR-221	154412.0900	135297.3400	0.1907	1.07E-171	up
gga-miR-106	65066.7700	55680.5300	0.2247	5.82E-108	up
gga-miR-200a	541.8800	53.5300	3.3396	6.11E-98	up
gga-miR-499	3145.2600	1636.5400	0.9425	1.48E-97	up
gga-miR-6557-3p	1018.9700	305.8900	1.7360	5.78E-85	up
gga-miR-383	1319.3600	512.3700	1.3646	2.03E-76	up
gga-miR-34b	589.0000	122.3600	2.2671	1.15E-70	up
gga-miR-34c	589.0000	122.3600	2.2671	1.15E-70	up
gga-miR-2188	151484.7600	138119.2200	0.1333	2.57E-66	up
gga-miR-128	107498.2800	97029.8800	0.1478	1.06E-62	up
gga-miR-24	30380.5900	25641.6400	0.2447	5.81E-62	up
gga-miR-200b	388.7400	53.5300	2.8604	4.43E-61	up
gga-miR-17-5p	90794.2600	81719.8400	0.1519	4.22E-57	up
gga-miR-6606-5p	665.5700	214.1300	1.6361	2.31E-51	up
gga-miR-214	43191.3300	37778.0300	0.1932	2.13E-50	up
gga-miR-1664-3p	1030.7500	451.2000	1.1919	2.19E-48	up
gga-miR-29b	30498.3900	26528.7400	0.2012	1.54E-39	up
gga-miR-27b	229915.9200	214952.3800	0.0971	1.74E-38	up
gga-miR-6552-3p	164.9200	0.0000	8.3656	5.15E-34	up
gga-miR-1744-3p	223.8200	38.2400	2.5492	1.47E-31	up
gga-miR-551-3p	200.2600	30.5900	2.7107	2.67E-30	up
gga-miR-20a	65325.9300	59511.8600	0.1345	5.90E-30	up
gga-miR-32	16079.6800	13902.9300	0.2098	1.47E-23	up
gga-miR-1416-5p	1372.3700	894.7400	0.6171	2.15E-20	up
gga-miR-6557-5p	235.6000	76.4700	1.6234	1.01E-18	up
gga-miR-153	20691.5500	18376.6400	0.1712	1.46E-18	up
gga-miR-23b	85858.4500	79938.0000	0.1031	3.88E-18	up
gga-miR-1684b-3p	1148.5500	741.8000	0.6307	5.87E-18	up
gga-miR-33	6149.1500	5070.2100	0.2783	1.15E-17	up
gga-miR-1684a-3p	1272.2400	848.8600	0.5838	3.49E-17	up
gga-miR-218	2273.5400	1690.0700	0.4279	1.58E-16	up
gga-miR-148a	1334325.1800	1284575.2600	0.0548	1.74E-15	up
gga-miR-449a	70.6800	7.6500	3.2078	2.54E-13	up

gga-miR-1559	25821.7300	23523.3200	0.1345	1.64E-12	up
gga-miR-1655	217.9300	91.7700	1.2478	6.84E-12	up
gga-miR-365	1896.5800	1460.6500	0.3768	6.00E-11	up
gga-miR-449b-5p	58.9000	7.6500	2.9447	1.83E-10	up
gga-miR-449c-5p	106.0200	30.5900	1.7932	3.00E-10	up
gga-miR-15a	179756.7300	178313.8200	0.0116	6.85E-09	up
gga-miR-1552-5p	907.0600	650.0300	0.4807	7.29E-09	up
gga-miR-3525	465.3100	298.2500	0.6417	4.12E-08	up
gga-miR-3531	29.4500	0.0000	5.8802	6.47E-08	up
gga-miR-196	241.4900	130.0100	0.8933	8.20E-08	up
gga-miR-429	376.9600	237.0700	0.6691	3.06E-07	up
gga-miR-10a-3p	9365.0900	8419.7600	0.1535	5.33E-07	up
gga-let-7d	3015.6800	2554.2200	0.2396	8.03E-07	up
gga-miR-1648-5p	23.5600	0.0000	5.5583	1.54E-06	up
gga-miR-3532	23.5600	0.0000	5.5583	1.54E-06	up
gga-miR-6552-5p	23.5600	0.0000	5.5583	1.54E-06	up
gga-miR-2964	136011.7500	134807.9100	0.0128	1.66E-06	up
gga-miR-1782	294.5000	183.5400	0.6822	4.48E-06	up
gga-miR-1666	17.6700	0.0000	5.1432	4.23E-05	up
gga-miR-206	17.6700	0.0000	5.1432	4.23E-05	up
gga-miR-6677-5p	17.6700	0.0000	5.1432	4.23E-05	up
gga-miR-460b-5p	806.9300	627.0800	0.3638	6.04E-05	up
gga-miR-103	189492.8900	186649.4500	0.0218	0.000170965	up
gga-miR-137	94.2400	45.8800	1.0385	0.000174111	up
gga-miR-1306	371.0700	267.6600	0.4713	0.000422017	up
gga-miR-1729-5p	3687.1400	3280.7200	0.1685	0.000504673	up
gga-miR-2130	29.4500	7.6500	1.9447	0.000695053	up
gga-miR-6568-3p	29.4500	7.6500	1.9447	0.000695053	up
gga-miR-21	2604101.9200	4456262.3700	-0.7750	0	down
gga-miR-22-3p	2389181.9200	3014197.0900	-0.3353	0	down
gga-miR-146c-5p	1645422.9000	2124455.7400	-0.3686	0	down
gga-miR-222b-3p	49805.7900	103553.0900	-1.0560	0	down
gga-miR-142-3p	470663.5500	571113.5200	-0.2791	0	down
gga-miR-451	646273.7300	757885.3300	-0.2298	0	down
gga-miR-155	121828.6400	168379.8800	-0.4669	0	down
gga-miR-144	33785.0100	56537.0300	-0.7428	0	down
gga-miR-146a	290082.2200	342862.3600	-0.2412	0	down
gga-miR-142-5p	3575096.9200	3622805.6900	-0.0191	0	down
gga-miR-30a-5p	3959377.8100	3984656.4800	-0.0092	0	down
gga-miR-30e	2745756.2800	2781204.5500	-0.0185	0	down
gga-miR-199-5p	148180.4700	169687.5800	-0.1955	0	down
gga-miR-140-3p	455396.6800	486181.7900	-0.0944	0	down
gga-miR-147	9553.5700	16342.4400	-0.7745	0	down
gga-miR-146c-3p	8440.3600	14713.5500	-0.8018	0	down
gga-miR-100	494105.7300	518881.9600	-0.0706	0	down
gga-miR-99a-5p	357275.2700	377030.8200	-0.0776	8.16E-270	down
gga-miR-181a-3p	41500.9000	48231.9900	-0.2168	6.18E-156	down
gga-miR-222a	60201.6300	67564.5500	-0.1665	6.84E-144	down
gga-miR-456	51926.1900	58754.7700	-0.1782	3.07E-139	down
gga-miR-301b-3p	102179.6200	109525.6900	-0.1002	1.46E-110	down
gga-miR-301a	95459.1400	101939.4900	-0.0948	1.71E-95	down
gga-miR-222b-5p	4982.9400	6829.1000	-0.4547	7.54E-76	down
gga-miR-9-3p	117.8000	565.9100	-2.2642	9.06E-73	down
gga-miR-215	4894.5900	6546.1500	-0.4195	1.07E-63	down
gga-miR-20b	20880.0300	23599.8000	-0.1767	7.61E-56	down
gga-miR-199-3p	493398.9300	493951.5200	-0.0016	3.65E-47	down
gga-miR-19b	246119.3000	249258.5000	-0.0183	5.42E-46	down
gga-miR-1662	2621.0500	3655.4400	-0.4799	5.93E-45	down
gga-miR-15b	159029.8400	161443.7100	-0.0217	1.32E-33	down
gga-miR-1769-3p	965.9600	1537.1200	-0.6702	2.70E-33	down
gga-miR-130a	91129.9900	93596.2100	-0.0385	2.70E-31	down
gga-miR-130c-3p	99411.3200	101679.4800	-0.0325	3.40E-29	down
gga-miR-3523	1030.7500	1567.7100	-0.6050	8.10E-29	down
gga-miR-19a	234463.0000	235546.7600	-0.0067	1.63E-28	down

gga-miR-1769-5p	347.5100	672.9700	-0.9535	4.19E-26	down
gga-miR-15c-5p	223631.3000	224458.0700	-0.0053	8.92E-26	down
gga-miR-130c-5p	600.7800	1001.8100	-0.7377	1.07E-25	down
gga-miR-194	176.7000	412.9600	-1.2247	9.89E-24	down
gga-miR-16	229821.6900	230208.8900	-0.0024	2.43E-23	down
gga-miR-133c	15690.9400	16984.8200	-0.1143	1.41E-21	down
gga-miR-1674	259.1600	512.3700	-0.9833	4.29E-21	down
gga-miR-133a	17092.7600	18384.2900	-0.1051	9.06E-21	down
gga-miR-99a-3p	2768.3000	3418.3800	-0.3043	2.98E-20	down
gga-miR-34a	3115.8100	3785.4500	-0.2809	1.13E-19	down
gga-miR-1563	624.3400	963.5700	-0.6261	3.80E-19	down
gga-miR-133b	15514.2400	16694.2200	-0.1058	3.94E-19	down
gga-miR-146b-3p	2238.2000	2806.5900	-0.3265	8.80E-19	down
gga-miR-193b	24602.5100	25817.5300	-0.0695	4.18E-17	down
gga-miR-458a-3p	16291.7200	17359.5400	-0.0916	1.72E-16	down
gga-miR-1329-5p	371.0700	619.4400	-0.7393	2.15E-16	down
gga-miR-16c	28083.4900	29151.7900	-0.0539	1.86E-14	down
gga-miR-6546-5p	23.5600	99.4200	-2.0772	1.81E-12	down
gga-miR-3536	58.9000	152.9500	-1.3767	4.53E-11	down
gga-miR-6649-5p	5.8900	53.5300	-3.1840	9.30E-11	down
gga-miR-193a	2191.0800	2561.8700	-0.2256	7.26E-10	down
gga-miR-3535	0.0000	30.5900	-5.9350	2.24E-08	down
gga-miR-454-3p	25474.2300	26024.0100	-0.0308	6.52E-08	down
gga-miR-490	907.0600	1124.1600	-0.3096	1.54E-07	down
gga-miR-190	1666.8700	1934.7900	-0.2150	3.39E-07	down
gga-miR-1756a	259.1600	382.3700	-0.5611	5.07E-07	down
gga-miR-183	4252.5800	4619.0100	-0.1192	5.56E-07	down
gga-miR-6553-3p	200.2600	305.8900	-0.6111	1.48E-06	down
gga-miR-1452	0.0000	22.9400	-5.5198	1.53E-06	down
gga-miR-1797	0.0000	22.9400	-5.5198	1.53E-06	down
gga-miR-1668-3p	53.0100	114.7100	-1.1137	1.65E-06	down
gga-miR-18b	8446.2500	8832.7200	-0.0645	4.71E-06	down
gga-miR-18a	9088.2600	9444.5100	-0.0555	1.63E-05	down
gga-miR-6631-5p	147.2500	229.4200	-0.6397	1.67E-05	down
gga-miR-6548-5p	70.6800	130.0100	-0.8792	2.73E-05	down
gga-miR-6542-3p	88.3500	152.9500	-0.7918	2.91E-05	down
gga-miR-1742	5.8900	30.5900	-2.3767	3.76E-05	down
gga-miR-1698	23.5600	61.1800	-1.3767	4.80E-05	down
gga-miR-1712-3p	23.5600	61.1800	-1.3767	4.80E-05	down
gga-miR-1458	29.4500	68.8300	-1.2248	8.01E-05	down
gga-miR-1585	0.0000	15.2900	-4.9345	0.000124084	down
gga-miR-1587	0.0000	15.2900	-4.9345	0.000124084	down
gga-miR-1626-5p	0.0000	15.2900	-4.9345	0.000124084	down
gga-miR-1668-5p	0.0000	15.2900	-4.9345	0.000124084	down
gga-miR-1699	0.0000	15.2900	-4.9345	0.000124084	down
gga-miR-6614-3p	0.0000	15.2900	-4.9345	0.000124084	down
gga-miR-138	530.1000	650.0300	-0.2942	0.000170237	down
gga-miR-1728-3p	17.6700	45.8800	-1.3766	0.000496471	down

[a]"up" means miRNA was up-regulated in ALV-J infected compared to uninfected chicken spleens.
"down" means miRNA was down-regulated in ALV-J infected compared to uninfected chicken spleens.

Table S2. Differential expression analysis of genes in AVL-J infected compared with uninfected chicken spleens using DEG.

Ensemble id	Wikigenes	ALV-J infected spleens (WRR+) FPKM	Uninfected spleens (WRR−) FPKM	log2 Ratio(WRR+/WRR−)	q value	Regualtion[a]
ENSGALG00000003493	RPS27L	6308.7100	3976.3100	0.6659	0.0220731	up
ENSGALG00000002157	RPS17L	1814.4900	1176.1700	0.6255	0.0280457	up
ENSGALG00000010336	LOC422509	2524.1200	1004.2100	1.3297	5.27E-07	up
ENSGALG00000024906	U3	1676.1600	965.8200	0.7953	0.0437163	up
ENSGALG00000009963	LYZ	1433.3100	617.0750	1.2158	1.23E-08	up
ENSGALG00000011351	HSP90AA1	1016.7500	616.8060	0.7211	0.0125173	up
ENSGALG00000011740	MGP	883.6940	395.8800	1.1585	5.96E-07	up
ENSGALG00000011888	TGM4	843.3320	355.8600	1.2448	0.000728876	up
ENSGALG00000012119	MARCO	722.0030	294.8210	1.2922	0	up
ENSGALG00000024011	LCN8	273.5510	170.8440	0.6791	0.0120618	up
ENSGALG00000011511	CKB	200.7020	139.0930	0.5290	0.0422742	up
ENSGALG00000011529	NAAA	269.2540	139.0210	0.9537	5.44E-07	up
ENSGALG00000012786	SPIC	337.9230	129.8490	1.3799	6.11E-09	up
ENSGALG00000015183	RAB13	287.6880	120.6020	1.2543	9.42E-08	up
ENSGALG00000013071	CALD1	157.1000	109.1120	0.5259	0.0152297	up
ENSGALG00000001164	CR2	362.1960	105.6140	1.7780	0	up
ENSGALG00000006520	MYH11	145.9010	99.5936	0.5509	0.000114137	up
ENSGALG00000001520	GGA.42342	156.8590	98.0308	0.6782	0.0422132	up
ENSGALG00000000318	CSRP1	147.7800	94.9176	0.6387	0.0470582	up
ENSGALG00000011916	SREBF2	154.9140	93.3359	0.7310	0.00118533	up
ENSGALG00000011214	SERPINH1	139.9780	87.3562	0.6802	0.0218924	up
ENSGALG00000009163	S100A11	142.7400	75.6869	0.9153	0.0206325	up
ENSGALG00000011446	TNFAIP2	110.7420	71.6364	0.6284	0.0440577	up
ENSGALG00000001446	GSN	119.9340	68.4778	0.8085	3.41E-05	up
ENSGALG00000004677	VSIG4	160.3790	67.0184	1.2589	8.34E-05	up
ENSGALG00000005060	ENG	106.8560	64.7816	0.7220	0.0020994	up
ENSGALG00000002667	OLFML2B	92.1451	56.8475	0.6968	0.0180725	up
ENSGALG00000015409	PROS1	102.4680	56.7780	0.8518	0.00103892	up
ENSGALG00000005069	PTGS2	80.0275	51.0210	0.6494	0.027382	up
ENSGALG00000018702	novel gene	103.5560	45.1726	1.1969	0.0349361	up
ENSGALG00000000227	DPYSL2	69.4370	45.1659	0.6205	0.020673	up
ENSGALG00000013218	C3AR	73.1992	44.1166	0.7305	0.0229212	up
ENSGALG00000013112	RERGL	102.7280	43.9204	1.2259	0.00152603	up
ENSGALG00000002829	SCARF1	70.3520	41.5145	0.7610	0.010168	up
ENSGALG00000023655	novel gene	81.7476	41.0600	0.9934	0.0216881	up
ENSGALG00000016292	COL21A1	77.6023	40.2982	0.9454	0.000154369	up
ENSGALG00000023948	novel gene	148.8730	37.2186	2.0000	6.48E-09	up
ENSGALG00000009641	COL1A2	60.9794	37.0589	0.7185	0.000583141	up
ENSGALG00000009626	THBS1	71.0634	36.2270	0.9720	7.06E-05	up
ENSGALG00000003876	TIMD4	85.5456	34.7797	1.2984	8.53E-07	up
ENSGALG00000012504	NGFR	92.3428	30.1858	1.6131	0.000261887	up
ENSGALG00000008757	CNRIP1	56.1778	29.0769	0.9501	0.0451103	up
ENSGALG00000008593	ATP1B4	49.2532	24.0850	1.0321	0.00184808	up
ENSGALG00000005554	ADIPOQ	42.1221	22.6908	0.8925	0.0196632	up
ENSGALG00000023528	EDN3	51.7529	21.1925	1.2881	0.00273177	up
ENSGALG00000002452	AQP3	61.3798	20.9872	1.5483	2.72E-06	up
ENSGALG00000008293	novel gene	35.5933	19.8467	0.8427	0.00663609	up
ENSGALG00000015001	LAMA4	31.0764	18.3277	0.7618	0.0072436	up
ENSGALG00000006563	SEM3D	47.0204	17.9789	1.3870	2.49E-07	up
ENSGALG00000010857	DAB1	28.7618	16.1637	0.8314	0.0087455	up
ENSGALG00000022907	novel gene	53.9263	15.3990	1.8082	0.0243658	up
ENSGALG00000016195	COL22A1	33.0758	14.9105	1.1494	5.32E-06	up
ENSGALG00000017393	PKDCC	32.4038	14.8473	1.1260	0.0140127	up
ENSGALG00000022805	novel gene	48.7130	14.6226	1.7361	5.99E-06	up
ENSGALG00000006802	LAMB2	23.9391	14.3548	0.7378	0.00904055	up
ENSGALG00000006172	ABCC8	22.3122	12.7909	0.8027	0.0130886	up
ENSGALG00000013244	ABCC9	23.0500	12.1752	0.9208	0.00175913	up
ENSGALG00000016411	COL14A1	22.9032	11.4089	1.0054	0.0120618	up
ENSGALG00000008367	MDK	30.1305	10.5791	1.5100	0.00347449	up
ENSGALG00000008735	BFSP1	27.1123	10.4658	1.3733	1.62E-05	up
ENSGALG00000010566	NPNT	25.4981	10.4204	1.2910	0.00199284	up

ENSGALG00000014835	C7	18.4332	9.6819	0.9289	0.0247254	up
ENSGALG00000014815	EMILIN2	19.2061	9.6800	0.9885	0.0110264	up
ENSGALG00000007222	UTS2D	39.5212	9.4812	2.0595	2.07E-06	up
ENSGALG00000015123	ZDHHC23	21.5787	9.2607	1.2204	9.77E-06	up
ENSGALG00000011078	PTPN13	16.9784	8.0240	1.0813	1.98E-05	up
ENSGALG00000010680	TTC7B	14.8870	7.6635	0.9580	0.0265443	up
ENSGALG00000009429	RXFP1	17.4894	6.8248	1.3576	0.000914608	up
ENSGALG00000016511	GPR64	18.6512	6.7716	1.4617	9.47E-05	up
ENSGALG00000007617	ANO1	14.7669	6.7381	1.1319	0.00836706	up
ENSGALG00000016575	GPM6B	16.9083	6.6464	1.3471	0.0143089	up
ENSGALG00000006705	MYOF	13.0386	6.6121	0.9796	0.00106217	up
ENSGALG00000002757	novel gene	25.2330	6.1487	2.0370	0.00292304	up
ENSGALG00000000994	RAB42	23.0524	6.1191	1.9135	0.00377855	up
ENSGALG00000005024	CCDC48	14.7045	5.5798	1.3980	0.0251733	up
ENSGALG00000015966	CTNNA2	13.1785	4.9792	1.4042	0.00190025	up
ENSGALG00000012083	GPR141	14.8779	4.4127	1.7534	0.00901941	up
ENSGALG00000017166	RAB39A	15.8065	4.0366	1.9693	0.0203131	up
ENSGALG00000016220	WISP1	15.6804	3.3687	2.2187	6.44E-05	up
ENSGALG00000010152	TSPAN8	13.7763	3.3329	2.0473	0.00136607	up
ENSGALG00000008509	SEMA3E	13.1018	3.0001	2.1267	7.69E-07	up
ENSGALG00000020151	NMU	12.4426	2.6840	2.2128	0.00170001	up
ENSGALG00000000827	SF3A2	138.2250	0.0000	1.79769e+308	1.21E-09	up
ENSGALG00000014922	ROCK1	69.1777	0.0000	1.79769e+308	2.67E-24	up
ENSGALG00000024367	novel gene	47.0575	0.0000	1.79769e+308	1.87E-08	up
ENSGALG00000007512	MORC2	33.9424	0.0000	1.79769e+308	5.32E-23	up
ENSGALG00000021247	CNEP1R1	33.8189	0.0000	1.79769e+308	0.00288836	up
ENSGALG00000024167	ISG20L2	22.5787	0.0000	1.79769e+308	1.24E-07	up
ENSGALG00000003874	HEATR3	22.1763	0.0000	1.79769e+308	2.63E-16	up
ENSGALG00000002496	LL	11.4608	0.0000	1.79769e+308	0.000322686	up
ENSGALG00000007468	HBAA	652.7320	6803.1000	-3.3816	0	down
ENSGALG00000017345	HBG1	675.7900	6346.2000	-3.2312	0	down
ENSGALG00000007463	HBAD	165.4300	3322.7900	-4.3281	0	down
ENSGALG00000022703	HBG2	118.3530	1015.5600	-3.1011	0	down
ENSGALG00000003196	ANG	444.1060	995.9640	-1.1652	1.82E-10	down
ENSGALG00000016152	LY6E	399.6370	719.0530	-0.8474	0.00299017	down
ENSGALG00000013575	ISG12-2	193.5610	716.6120	-1.8884	0	down
ENSGALG00000005946	novel gene	238.8340	438.9510	-0.8781	0.0444808	down
ENSGALG00000007651	STAT1	204.7630	424.8880	-1.0531	3.26E-06	down
ENSGALG00000016829	LAMP1	218.6680	328.0010	-0.5850	0.014471	down
ENSGALG00000015862	CA2	62.5318	314.6100	-2.3309	0	down
ENSGALG00000002902	novel gene	174.3750	303.5410	-0.7997	0.00298581	down
ENSGALG00000006384	IFIT5	68.8033	289.7040	-2.0740	0	down
ENSGALG00000006785	IRF1	187.6750	283.5330	-0.5953	0.0194019	down
ENSGALG00000008835	SAMD13	200.0570	278.0830	-0.4751	0.0451103	down
ENSGALG00000010811	LGMN	167.4380	265.7650	-0.6665	0.0324755	down
ENSGALG00000023370	novel gene	125.0920	260.2930	-1.0571	0.0220731	down
ENSGALG00000012610	CTSL2	134.3080	254.6710	-0.9231	2.34E-07	down
ENSGALG00000017186	BIRC2	147.5700	247.7530	-0.7475	8.34E-05	down
ENSGALG00000012357	CXCR4	133.0090	243.9030	-0.8748	0.000633073	down
ENSGALG00000012313	H1F0	112.5720	218.2920	-0.9554	0.00377855	down
ENSGALG00000021627	IFI27L2	72.3068	217.0710	-1.5860	2.12E-06	down
ENSGALG00000011826	PLBD1	86.9448	153.7670	-0.8226	0.00199253	down
ENSGALG00000015440	RCSD1	100.7590	153.2840	-0.6053	0.040992	down
ENSGALG00000006378	LIPA	95.9460	152.1700	-0.6654	0.0268453	down
ENSGALG00000012072	PARP15	80.3501	134.4480	-0.7427	0.0213684	down
ENSGALG00000001629	LOC417345	78.4619	133.9960	-0.7721	0.0115828	down
ENSGALG00000012784	TXNDC5	79.2419	128.4070	-0.6964	0.0159887	down
ENSGALG00000011153	LY75	78.0323	114.8420	-0.5575	0.00747925	down
ENSGALG00000003597	AK2	63.2963	108.7230	-0.7805	0.0141272	down
ENSGALG00000000720	GBP7	66.3665	107.1940	-0.6917	0.0180635	down
ENSGALG00000010476	BNIP3	59.8149	105.0390	-0.8123	0.0103493	down
ENSGALG00000002329	CCL1	47.6414	103.4110	-1.1181	0.0206325	down
ENSGALG00000019795	novel gene	38.1212	102.2540	-1.4235	7.59E-06	down
ENSGALG00000008257	MERTK	55.3470	100.0430	-0.8540	0.000721104	down
ENSGALG00000014477	CD4	55.0231	98.3413	-0.8378	0.00103327	down
ENSGALG00000002549	RGS1	52.0715	96.0099	-0.8827	0.00837528	down
ENSGALG00000012480	NMI	56.2541	94.0707	-0.7418	0.0215854	down

ENSGALG00000006351	FAS	60.1003	92.4175	-0.6208	0.0276367	down
ENSGALG00000004614	NPL	38.4291	92.2258	-1.2630	2.53E-06	down
ENSGALG00000002783	SLC43A2	41.6474	91.7330	-1.1392	6.60E-06	down
ENSGALG00000003584	KPNA2	51.9748	90.9272	-0.8069	0.00403041	down
ENSGALG00000004859	ZNFX1	37.3040	89.2360	-1.2583	7.71E-08	down
ENSGALG00000016173	UBASH3A	57.4399	86.5401	-0.5913	0.0224748	down
ENSGALG00000001558	MOV10	45.8631	81.4093	-0.8279	0.00212864	down
ENSGALG00000019141	LYG2	12.4333	81.3108	-2.7092	8.63E-12	down
ENSGALG00000024350	novel gene	35.7806	80.8050	-1.1753	0.000583141	down
ENSGALG00000014811	SMCHD1	59.2765	80.5103	-0.4417	0.040992	down
ENSGALG00000016099	novel gene	40.1436	79.5881	-0.9874	0.0233684	down
ENSGALG00000007447	TUBB1	25.7398	79.5080	-1.6271	3.48E-12	down
ENSGALG00000016964	EPSTI1	33.9334	79.1987	-1.2228	0.000721499	down
ENSGALG00000006425	SLC6A6	50.6603	78.9541	-0.6402	0.0442082	down
ENSGALG00000016142	MX1	9.4955	74.9547	-2.9807	0	down
ENSGALG00000013057	USP18	25.9695	74.6632	-1.5236	5.33E-10	down
ENSGALG00000006988	RNF213	30.9880	70.4357	-1.1846	1.51E-05	down
ENSGALG00000011733	CCR2	36.8635	68.5709	-0.8954	0.0256369	down
ENSGALG00000013101	novel gene	17.8322	68.2274	-1.9359	3.83E-11	down
ENSGALG00000016398	CMPK2	13.2637	67.3130	-2.3434	1.47E-05	down
ENSGALG00000009240	NFE2L2	40.7554	63.9418	-0.6498	0.0194306	down
ENSGALG00000010629	ZCCHC11	36.7354	62.2242	-0.7603	0.00521562	down
ENSGALG00000020220	novel gene	16.6433	61.9700	-1.8966	3.05E-07	down
ENSGALG00000002431	CFH	40.0538	61.5802	-0.6205	0.0449451	down
ENSGALG00000016721	PLCXD1	27.0898	61.1129	-1.1737	0.000339995	down
ENSGALG00000009479	SAMD9L	15.1032	61.0749	-2.0157	0	down
ENSGALG00000020219	novel gene	15.3669	60.6152	-1.9799	5.33E-10	down
ENSGALG00000021393	novel gene	22.8198	60.1176	-1.3975	0.000119819	down
ENSGALG00000011955	CX3CR1	29.6454	57.9316	-0.9665	0.0233638	down
ENSGALG00000012830	IRF4	37.9190	57.3401	-0.5966	0.0488699	down
ENSGALG00000015712	SLBP	22.2286	55.0994	-1.3096	1.66E-05	down
ENSGALG00000015900	novel gene	7.7437	54.7825	-2.8226	9.67E-06	down
ENSGALG00000014453	FAM46C	17.9314	54.3440	-1.5996	1.03E-05	down
ENSGALG00000011089	IFIH1	25.6737	54.0603	-1.0743	0.000119819	down
ENSGALG00000012173	LGALS3	25.8387	53.9854	-1.0630	0.010168	down
ENSGALG00000004727	ULK2	29.0352	53.3217	-0.8769	0.00103315	down
ENSGALG00000013911	ZC3HAV1	30.5611	51.9723	-0.7660	0.00521562	down
ENSGALG00000021467	CX3CL1	20.8852	51.5959	-1.3048	0.0025114	down
ENSGALG00000008621	NRP2	33.5538	51.5064	-0.6183	0.0143089	down
ENSGALG00000006126	COL6A2	27.5564	50.7635	-0.8814	0.000810331	down
ENSGALG00000012057	TRANK1	22.5373	50.0981	-1.1524	9.02E-07	down
ENSGALG00000007485	TFRC	26.5740	49.2362	-0.8897	0.00387048	down
ENSGALG00000010149	FTSJD2	25.3941	48.9693	-0.9474	0.00174196	down
ENSGALG00000001561	MXRA8	27.8420	48.3847	-0.7973	0.0191329	down
ENSGALG00000003578	FN1	16.9758	47.2428	-1.4766	4.30E-12	down
ENSGALG00000003527	ARRDC2	25.5533	46.8829	-0.8756	0.0136235	down
ENSGALG00000001395	novel gene	17.1639	46.8281	-1.4480	0.0180725	down
ENSGALG00000011798	HEBP1	24.5871	45.9593	-0.9025	0.00219329	down
ENSGALG00000004728	RGL1	30.3201	45.7610	-0.5938	0.0363964	down
ENSGALG00000014127	TXK	25.4733	44.9575	-0.8196	0.0454993	down
ENSGALG00000017302	SLCO2B1	26.1008	44.9451	-0.7841	0.0323507	down
ENSGALG00000008666	CTLA4	17.8859	44.5663	-1.3171	0.00578906	down
ENSGALG00000016786	IL18R1	22.0173	44.3116	-1.0090	0.00283805	down
ENSGALG00000006267	TPX2	25.8939	44.2807	-0.7741	0.0159887	down
ENSGALG00000016399	SNTB1	23.4159	43.1244	-0.8810	7.60E-05	down
ENSGALG00000003212	TSPO2	4.4931	42.8168	-3.2524	5.80E-12	down
ENSGALG00000008839	ATP11B	0.0000	42.3410	-1.79769e+308	1.68E-30	down
ENSGALG00000021226	novel gene	17.0353	42.2756	-1.3113	0.00415409	down
ENSGALG00000010560	EIF2AK2	23.2727	42.1265	-0.8561	0.00496684	down
ENSGALG00000011046	SNX10	25.6699	41.7870	-0.7030	0.0348776	down
ENSGALG00000008669	CD28	19.0690	40.5967	-1.0901	0.0174143	down
ENSGALG00000005974	COL6A1	17.3967	40.0782	-1.2040	8.24E-07	down
ENSGALG00000003144	TRIM25	20.7429	39.1480	-0.9163	0.0249714	down
ENSGALG00000004205	SOAT1	21.5510	38.7060	-0.8448	0.0301956	down
ENSGALG00000001882	TNFRSF18	22.0424	38.1007	-0.7895	0.0156132	down
ENSGALG00000006489	CHCHD3	0.0000	38.0321	-1.79769e+308	1.81E-07	down
ENSGALG00000011149	PLA2R1	18.8651	37.6376	-0.9965	8.34E-05	down

ENSGALG00000014658	ARRDC3	18.2535	37.0040	-1.0195	0.0176332	down
ENSGALG00000015433	ABCA1	18.4027	36.8680	-1.0025	3.26E-06	down
ENSGALG00000005845	SLC7A5	18.1089	34.8630	-0.9450	0.000280909	down
ENSGALG00000005022	GP9	12.2173	34.0696	-1.4796	0.0243095	down
ENSGALG00000016400	RSAD2	5.9696	33.9855	-2.5092	7.71E-08	down
ENSGALG00000013993	VNN1	13.0623	33.0309	-1.3384	0.000247969	down
ENSGALG00000003701	SGMS1	0.0000	32.1293	-1.79769e+308	2.75E-15	down
ENSGALG00000013548	GZMA	15.9436	31.8832	-0.9998	0.0340161	down
ENSGALG00000015681	novel gene	17.7092	31.1504	-0.8148	0.013859	down
ENSGALG00000023373	novel gene	9.7616	31.0929	-1.6714	0.000938031	down
ENSGALG00000005321	LAMA5	15.5551	30.0344	-0.9492	0.000119819	down
ENSGALG00000014716	IL6ST	17.0215	29.8613	-0.8109	0.0273944	down
ENSGALG00000003920	OCRL	0.0000	28.6494	-1.79769e+308	1.22E-20	down
ENSGALG00000019609	TRANK1	8.5833	27.8523	-1.6982	0.00880009	down
ENSGALG00000006110	PLK1	15.2651	27.4533	-0.8467	0.035588	down
ENSGALG00000003288	TARS	0.0000	27.1526	-1.79769e+308	9.47E-20	down
ENSGALG00000007113	TNC	17.6498	27.1388	-0.6207	0.0213684	down
ENSGALG00000002445	KIAA0319L	0.0000	26.6076	-1.79769e+308	1.46E-24	down
ENSGALG00000006790	KIF11	15.1507	26.2100	-0.7907	0.0213877	down
ENSGALG00000000873	GGA.47157	8.9190	26.1112	-1.5497	0.0110054	down
ENSGALG00000009728	SLC25A13	0.0000	25.8201	-1.79769e+308	3.49E-22	down
ENSGALG00000002099	PFKFB4	10.5021	25.4462	-1.2768	0.00663609	down
ENSGALG00000015820	CA13	6.6992	25.3456	-1.9197	0.000771485	down
ENSGALG00000016651	TDH	7.7877	24.1951	-1.6355	0.000220576	down
ENSGALG00000012484	RIF1	16.1349	24.1276	-0.5805	0.029507	down
ENSGALG00000006138	HELZ2	10.2135	22.8046	-1.1588	3.71E-06	down
ENSGALG00000007575	PPP4R1L	0.0000	22.2140	-1.79769e+308	3.57E-22	down
ENSGALG00000010649	CNST	10.6520	22.0176	-1.0475	0.0192507	down
ENSGALG00000004468	FAM82A2	0.0000	20.2770	-1.79769e+308	2.38E-11	down
ENSGALG00000001215	RHD	5.6708	19.3345	-1.7696	0.00111812	down
ENSGALG00000012404	ERO1L	0.0000	18.8391	-1.79769e+308	8.62E-12	down
ENSGALG00000006236	TPH1	4.7709	18.5534	-1.9593	6.44E-05	down
ENSGALG00000011954	CCR8	7.2410	18.4534	-1.3496	0.00121925	down
ENSGALG00000019325	novel gene	7.3833	18.4047	-1.3177	0.040992	down
ENSGALG00000010961	IGF2BP	10.5746	18.0823	-0.7740	0.0410446	down
ENSGALG00000014935	GREB1L	8.2978	17.7147	-1.0942	0.00017672	down
ENSGALG00000014236	ATP8A1	9.6803	17.1941	-0.8288	0.040992	down
ENSGALG00000023815	novel gene	5.3362	17.1379	-1.6833	0.0437163	down
ENSGALG00000016324	GSTA3	3.9687	16.6754	-2.0710	0.00149743	down
ENSGALG00000022693	novel gene	3.1675	15.8860	-2.3263	0.00209185	down
ENSGALG00000001233	RHD	4.9137	15.8336	-1.6881	0.0079315	down
ENSGALG00000002630	ZNF76	0.0000	15.6108	-1.79769e+308	2.92E-12	down
ENSGALG00000005792	ABCG2	0.0000	15.5463	-1.79769e+308	2.51E-12	down
ENSGALG00000014722	LOC769174	4.8064	15.2419	-1.6650	0.0335174	down
ENSGALG00000009639	DDX60	2.0675	14.6679	-2.8267	2.21E-13	down
ENSGALG00000000378	SLC25A37	3.5735	14.2619	-1.9968	0.0020994	down
ENSGALG00000024352	novel gene	3.0905	14.1275	-2.1926	0.00996502	down
ENSGALG00000016785	IL1RL1	5.4756	13.7486	-1.3282	0.0196632	down
ENSGALG00000016684	RHAG	1.2640	13.5782	-3.4252	2.31E-08	down
ENSGALG00000009552	novel gene	4.4956	12.7339	-1.5021	0.0268453	down
ENSGALG00000003386	SPTB	1.6386	12.5656	-2.9389	0	down
ENSGALG00000002338	ASPM	6.6821	12.0420	-0.8497	0.0256369	down
ENSGALG00000015469	IGSF3	5.9140	11.6918	-0.9833	0.0449451	down
ENSGALG00000016788	IL18RAP	4.3968	10.8898	-1.3084	0.0478141	down
ENSGALG00000020444	novel gene	2.8743	10.6854	-1.8944	0.0484265	down
ENSGALG00000010158	KANK1	4.8537	10.5986	-1.1267	0.010168	down
ENSGALG00000009700	PDK4	1.9545	10.1462	-2.3761	0.000218755	down

[a]"up" means miRNA was up-regulated in ALV-J infected compared to uninfected chicken spleens.
"down" means miRNA was down-regulated in ALV-J infected compared to uninfected chicken spleens.

Table S3. Differentially expressed miRNAs and theirs corresponding target genes with differential expression in AVL-J infected compared with uninfected chicken spleens.

miRNA-name	ALV-J infected spleens (WRR+) FPKM	Uninfected spleens (WRR−) FPKM	q value	Gene-name	ALV-J infected spleens (WRR+) FPKM	Uninfected spleens (WRR−) FPKM	q value	Correlation[a]
gga-miR-194	176.7	412.96	9.89E-24	HSP90AA1	1016.75	616.806	0.0125173	Negative
gga-miR-1329-5p	371.07	619.44	2.15E-16	HSP90AA1	1016.75	616.806	0.0125173	Negative
gga-miR-20b	20880.03	23599.8	7.61E-56	CKB	200.702	139.093	0.0422742	Negative
gga-miR-1458	29.45	68.83	8.01E-05	SPIC	337.923	129.849	6.11E-09	Negative
gga-miR-130c-3p	99411.32	101679.48	3.40E-29	MYH11	145.901	99.5936	0.000114137	Negative
gga-miR-130a	91129.99	93596.21	2.70E-31	MYH11	145.901	99.5936	0.000114137	Negative
gga-miR-454-3p	25474.23	26024.01	6.52E-08	MYH11	145.901	99.5936	0.000114137	Negative
gga-miR-1769-3p	965.96	1537.12	2.70E-33	MYH11	145.901	99.5936	0.000114137	Negative
gga-miR-1699	0	15.29	0.000124084	MYH11	145.901	99.5936	0.000114137	Negative
gga-miR-1587	0	15.29	0.000124084	CSRP1	147.78	94.9176	0.0470582	Negative
gga-miR-15b	159029.84	161443.71	1.32E-33	CSRP1	147.78	94.9176	0.0470582	Negative
gga-miR-16c	28083.49	29151.79	1.86E-14	CSRP1	147.78	94.9176	0.0470582	Negative
gga-miR-16	229821.69	230208.89	2.43E-23	CSRP1	147.78	94.9176	0.0470582	Negative
gga-miR-138	530.1	650.03	0.000170237	CSRP1	147.78	94.9176	0.0470582	Negative
gga-miR-1585	0	15.29	0.000124084	CSRP1	147.78	94.9176	0.0470582	Negative
gga-miR-1668-3p	53.01	114.71	1.65E-06	CSRP1	147.78	94.9176	0.0470582	Negative
gga-miR-138	530.1	650.03	0.000170237	SREBF2	154.914	93.3359	0.00118533	Negative
gga-miR-20b	20880.03	23599.8	7.61E-56	PTGS2	80.0275	51.021	0.027382	Negative
gga-miR-215	4894.59	6546.15	1.07E-63	PTGS2	80.0275	51.021	0.027382	Negative
gga-miR-30e	2745756.28	2781204.55	0	DPYSL2	69.437	45.1659	0.020673	Negative
gga-miR-144	33785.01	56537.03	0	RERGL	102.728	43.9204	0.00152603	Negative
gga-miR-19a	234463	235546.76	1.63E-28	THBS1	71.0634	36.227	7.06E-05	Negative
gga-miR-1458	29.45	68.83	8.01E-05	THBS1	71.0634	36.227	7.06E-05	Negative
gga-miR-144	33785.01	56537.03	0	THBS1	71.0634	36.227	7.06E-05	Negative
gga-miR-19b	246119.3	249258.5	5.42E-46	THBS1	71.0634	36.227	7.06E-05	Negative
gga-miR-18a	9088.26	9444.51	1.63E-05	THBS1	71.0634	36.227	7.06E-05	Negative
gga-miR-18b	8446.25	8832.72	4.71E-06	THBS1	71.0634	36.227	7.06E-05	Negative
gga-miR-3536	58.9	152.95	4.53E-11	TIMD4	85.5456	34.7797	8.53E-07	Negative
gga-miR-1769-3p	965.96	1537.12	2.70E-33	NGFR	92.3428	30.1858	0.000261887	Negative
gga-miR-133a	17092.76	18384.29	9.06E-21	NGFR	92.3428	30.1858	0.000261887	Negative
gga-miR-133c	15690.94	16984.82	1.41E-21	NGFR	92.3428	30.1858	0.000261887	Negative
gga-miR-133b	15514.24	16694.22	3.94E-19	NGFR	92.3428	30.1858	0.000261887	Negative
gga-miR-1698	23.56	61.18	4.80E-05	NGFR	92.3428	30.1858	0.000261887	Negative
gga-miR-1587	0	15.29	0.000124084	NGFR	92.3428	30.1858	0.000261887	Negative
gga-miR-140-3p	455396.68	486181.79	0	CNRIP1	56.1778	29.0769	0.0451103	Negative
gga-miR-144	33785.01	56537.03	0	CNRIP1	56.1778	29.0769	0.0451103	Negative
gga-miR-490	907.06	1124.16	1.54E-07	CNRIP1	56.1778	29.0769	0.0451103	Negative
gga-miR-1626-5p	0	15.29	0.000124084	ATP1B4	49.2532	24.085	0.00184808	Negative
gga-miR-3536	58.9	152.95	4.53E-11	EDN3	51.7529	21.1925	0.00273177	Negative
gga-miR-1452	0	22.94	1.53E-06	LAMA4	31.0764	18.3277	0.0072436	Negative
gga-miR-30e	2745756.28	2781204.55	0	LAMA4	31.0764	18.3277	0.0072436	Negative
gga-miR-99a-5p	357275.27	377030.82	8.16E-270	DAB1	28.7618	16.1637	0.0087455	Negative
gga-miR-100	494105.73	518881.96	0	DAB1	28.7618	16.1637	0.0087455	Negative
gga-miR-15b	159029.84	161443.71	1.32E-33	DAB1	28.7618	16.1637	0.0087455	Negative
gga-miR-16	229821.69	230208.89	2.43E-23	DAB1	28.7618	16.1637	0.0087455	Negative
gga-miR-16c	28083.49	29151.79	1.86E-14	DAB1	28.7618	16.1637	0.0087455	Negative
gga-miR-490	907.06	1124.16	1.54E-07	DAB1	28.7618	16.1637	0.0087455	Negative
gga-miR-456	51926.19	58754.77	3.07E-139	COL22A1	33.0758	14.9105	5.32E-06	Negative
gga-miR-15b	159029.84	161443.71	1.32E-33	PKDCC	32.4038	14.8473	0.0140127	Negative
gga-miR-16	229821.69	230208.89	2.43E-23	PKDCC	32.4038	14.8473	0.0140127	Negative
gga-miR-16c	28083.49	29151.79	1.86E-14	PKDCC	32.4038	14.8473	0.0140127	Negative
gga-miR-9-3p	117.8	565.91	9.06E-73	ABCC8	22.3122	12.7909	0.0130886	Negative
gga-miR-155	121828.64	168379.88	0	ABCC8	22.3122	12.7909	0.0130886	Negative
gga-miR-1769-3p	965.96	1537.12	2.70E-33	ABCC9	23.05	12.1752	0.00175913	Negative
gga-miR-1699	0	15.29	0.000124084	MDK	30.1305	10.5791	0.00347449	Negative
gga-miR-1585	0	15.29	0.000124084	BFSP1	27.1123	10.4658	1.62E-05	Negative
gga-miR-215	4894.59	6546.15	1.07E-63	C7	18.4332	9.68193	0.0247254	Negative
gga-miR-130c-3p	99411.32	101679.48	3.40E-29	ZDHHC23	21.5787	9.26066	9.77E-06	Negative
gga-miR-130a	91129.99	93596.21	2.70E-31	ZDHHC23	21.5787	9.26066	9.77E-06	Negative
gga-miR-454-3p	25474.23	26024.01	6.52E-08	ZDHHC23	21.5787	9.26066	9.77E-06	Negative
gga-miR-19a	234463	235546.76	1.63E-28	ZDHHC23	21.5787	9.26066	9.77E-06	Negative
gga-miR-19b	246119.3	249258.5	5.42E-46	ZDHHC23	21.5787	9.26066	9.77E-06	Negative
gga-miR-1712-3p	23.56	61.18	4.80E-05	PTPN13	16.9784	8.02404	1.98E-05	Negative
gga-miR-183	4252.58	4619.01	5.56E-07	TTC7B	14.887	7.66349	0.0265443	Negative
gga-miR-194	176.7	412.96	9.89E-24	TTC7B	14.887	7.66349	0.0265443	Negative
gga-miR-34a	3115.81	3785.45	1.13E-19	GPR64	18.6512	6.77161	9.47E-05	Negative
gga-miR-144	33785.01	56537.03	0	GPR64	18.6512	6.77161	9.47E-05	Negative
gga-miR-19b	246119.3	249258.5	5.42E-46	ANO1	14.7669	6.73814	0.00836706	Negative
gga-miR-19a	234463	235546.76	1.63E-28	ANO1	14.7669	6.73814	0.00836706	Negative
gga-miR-1626-5p	0	15.29	0.000124084	ANO1	14.7669	6.73814	0.00836706	Negative
gga-miR-215	4894.59	6546.15	1.07E-63	MYOF	13.0386	6.61211	0.00106217	Negative
gga-miR-133c	15690.94	16984.82	1.41E-21	MYOF	13.0386	6.61211	0.00106217	Negative
gga-miR-133a	17092.76	18384.29	9.06E-21	MYOF	13.0386	6.61211	0.00106217	Negative
gga-miR-34a	3115.81	3785.45	1.13E-19	MYOF	13.0386	6.61211	0.00106217	Negative
gga-miR-20b	20880.03	23599.8	7.61E-56	MYOF	13.0386	6.61211	0.00106217	Negative
gga-miR-144	33785.01	56537.03	0	SEMA3E	13.1018	3.00007	7.69E-07	Negative
gga-miR-215	4894.59	6546.15	1.07E-63	SEMA3E	13.1018	3.00007	7.69E-07	Negative
gga-miR-34a	3115.81	3785.45	1.13E-19	MORC2	33.9424	0	5.32E-23	Negative
gga-miR-9-3p	117.8	565.91	9.06E-73	LL	11.4608	0	0.000322686	Negative
gga-miR-15a	179756.73	178313.82	6.85E-09	ANG	444.106	995.964	1.82E-10	Negative
gga-miR-23b	85858.45	79938	3.88E-18	LAMP1	218.668	328.001	0.014471	Negative
gga-miR-153	20691.55	18376.64	1.46E-18	LAMP1	218.668	328.001	0.014471	Negative
gga-miR-218	2273.54	1690.07	1.58E-16	CA2	62.5318	314.61	0	Negative
gga-miR-23b	85858.45	79938	3.88E-18	CA2	62.5318	314.61	0	Negative
gga-miR-23b	85858.45	79938	3.88E-18	IRF1	187.675	283.533	0.0194019	Negative
gga-miR-2964	136011.75	134807.91	1.66E-06	IRF1	187.675	283.533	0.0194019	Negative
gga-miR-128	107498.28	97029.88	1.06E-62	SAMD13	200.057	278.083	0.0451103	Negative
gga-miR-23b	85858.45	79938	3.88E-18	SAMD13	200.057	278.083	0.0451103	Negative
gga-miR-211	18812.64	12564.64	7.28E-237	SAMD13	200.057	278.083	0.0451103	Negative

gga-miR-204	18812.64	12564.64	7.28E-237	SAMD13	200.057	278.083	0.0451103	Negative
gga-miR-24	30380.59	25641.64	5.81E-62	SAMD13	200.057	278.083	0.0451103	Negative
gga-miR-1782	294.5	183.54	4.48E-06	LGMN	167.438	265.765	0.0324755	Negative
gga-miR-23b	85858.45	79938	3.88E-18	CTSL2	134.308	254.671	2.34E-07	Negative
gga-miR-196	241.49	130.01	8.20E-08	BIRC2	147.57	247.753	8.34E-05	Negative
gga-miR-20a	65325.93	59511.86	5.90E-30	CXCR4	133.009	243.903	0.000633073	Negative
gga-miR-106	65066.77	55680.53	5.82E-108	CXCR4	133.009	243.903	0.000633073	Negative
gga-miR-23b	85858.45	79938	3.88E-18	CXCR4	133.009	243.903	0.000633073	Negative
gga-miR-221	154412.09	135297.34	1.07E-171	CXCR4	133.009	243.903	0.000633073	Negative
gga-miR-3531	29.45	0	6.47E-08	H1F0	112.572	218.292	0.00377855	Negative
gga-miR-1655	217.93	91.77	6.84E-12	PLBD1	86.9448	153.767	0.00199253	Negative
gga-miR-34b	589	122.36	1.15E-70	RCSD1	100.759	153.284	0.040992	Negative
gga-miR-429	376.96	237.07	3.06E-07	LIPA	95.946	152.17	0.0268453	Negative
gga-miR-200b	388.74	53.53	4.43E-61	LIPA	95.946	152.17	0.0268453	Negative
gga-miR-1655	217.93	91.77	6.84E-12	LOC417345	78.4619	133.996	0.0115828	Negative
gga-miR-1664-3p	1030.75	451.2	2.19E-48	TXNDC5	79.2419	128.407	0.0159887	Negative
gga-miR-30c	419880.02	323629.21	0	TXNDC5	79.2419	128.407	0.0159887	Negative
gga-miR-30b	89639.82	73254.19	2.08E-267	TXNDC5	79.2419	128.407	0.0159887	Negative
gga-miR-2964	136011.75	134807.91	1.66E-06	TXNDC5	79.2419	128.407	0.0159887	Negative
gga-miR-30d	2395549	2262888.5	1.05E-219	TXNDC5	79.2419	128.407	0.0159887	Negative
gga-miR-200b	388.74	53.53	4.43E-61	TXNDC5	79.2419	128.407	0.0159887	Negative
gga-miR-429	376.96	237.07	3.06E-07	TXNDC5	79.2419	128.407	0.0159887	Negative
gga-miR-2964	136011.75	134807.91	1.66E-06	GBP7	66.3665	107.194	0.0180635	Negative
gga-miR-223	114460.26	97374.01	2.84E-206	GBP7	66.3665	107.194	0.0180635	Negative
gga-miR-429	376.96	237.07	3.06E-07	GBP7	66.3665	107.194	0.0180635	Negative
gga-miR-200b	388.74	53.53	4.43E-61	GBP7	66.3665	107.194	0.0180635	Negative
gga-miR-33	6149.15	5070.21	1.15E-17	BNIP3	59.8149	105.039	0.0103493	Negative
gga-miR-15a	179756.73	178313.82	6.85E-09	BNIP3	59.8149	105.039	0.0103493	Negative
gga-miR-92	1955472.2	1805797.32	0	BNIP3	59.8149	105.039	0.0103493	Negative
gga-miR-26a	6688359.39	6171874.21	0	BNIP3	59.8149	105.039	0.0103493	Negative
gga-miR-27b	229915.92	214952.38	1.74E-38	RGS1	52.0715	96.0099	0.00837528	Negative
gga-miR-383	1319.36	512.37	2.03E-76	NMI	56.2541	94.0707	0.0215854	Negative
gga-miR-15a	179756.73	178313.82	6.85E-09	NMI	56.2541	94.0707	0.0215854	Negative
gga-miR-34b	589	122.36	1.15E-70	SLC43A2	41.6474	91.733	6.60E-06	Negative
gga-miR-29b	30498.39	26528.74	1.54E-39	SLC43A2	41.6474	91.733	6.60E-06	Negative
gga-miR-2964	136011.75	134807.91	1.66E-06	KPNA2	51.9748	90.9272	0.00403041	Negative
gga-miR-106	65066.77	55680.53	5.82E-108	KPNA2	51.9748	90.9272	0.00403041	Negative
gga-miR-20a	65325.93	59511.86	5.90E-30	KPNA2	51.9748	90.9272	0.00403041	Negative
gga-miR-26a	6688359.39	6171874.21	0	KPNA2	51.9748	90.9272	0.00403041	Negative
gga-miR-23b	85858.45	79938	3.88E-18	SMCHD1	59.2765	80.5103	0.040992	Negative
gga-miR-2964	136011.75	134807.91	1.66E-06	SMCHD1	59.2765	80.5103	0.040992	Negative
gga-miR-26a	6688359.39	6171874.21	0	TUBB1	25.7398	79.508	3.48E-12	Negative
gga-miR-137	94.24	45.88	0.000174111	SLC6A6	50.6603	78.9541	0.0442082	Negative
gga-miR-460b-5p	806.93	627.08	6.04E-05	MX1	9.4955	74.9547	0	Negative
gga-miR-449c-5p	106.02	30.59	3.00E-10	RNF213	30.988	70.4357	1.51E-05	Negative
gga-miR-449b-5p	58.9	7.65	1.83E-10	RNF213	30.988	70.4357	1.51E-05	Negative
gga-miR-34c	589	122.36	1.15E-70	RNF213	30.988	70.4357	1.51E-05	Negative
gga-miR-27b	229915.92	214952.38	1.74E-38	NFE2L2	40.7554	63.9418	0.0194306	Negative
gga-miR-153	20691.55	18376.64	1.46E-18	NFE2L2	40.7554	63.9418	0.0194306	Negative
gga-miR-26a	6688359.39	6171874.21	0	ZCCHC11	36.7354	62.2242	0.00521562	Negative
gga-miR-33	6149.15	5070.21	1.15E-17	ZCCHC11	36.7354	62.2242	0.00521562	Negative
gga-miR-211	18812.64	12564.64	7.28E-237	ZCCHC11	36.7354	62.2242	0.00521562	Negative
gga-miR-204	18812.64	12564.64	7.28E-237	ZCCHC11	36.7354	62.2242	0.00521562	Negative
gga-miR-30b	89639.82	73254.19	2.08E-267	CFH	40.0538	61.5802	0.0449451	Negative
gga-miR-30c	419880.02	323629.21	0	CFH	40.0538	61.5802	0.0449451	Negative
gga-miR-30d	2395549	2262888.5	1.05E-219	CFH	40.0538	61.5802	0.0449451	Negative
gga-miR-27b	229915.92	214952.38	1.74E-38	PLCXD1	27.0898	61.1129	0.000339995	Negative
gga-miR-20a	65325.93	59511.86	5.90E-30	PLCXD1	27.0898	61.1129	0.000339995	Negative
gga-miR-106	65066.77	55680.53	5.82E-108	PLCXD1	27.0898	61.1129	0.000339995	Negative
gga-miR-26a	6688359.39	6171874.21	0	PLCXD1	27.0898	61.1129	0.000339995	Negative
gga-miR-200b	388.74	53.53	4.43E-61	PLCXD1	27.0898	61.1129	0.000339995	Negative
gga-miR-429	376.96	237.07	3.06E-07	PLCXD1	27.0898	61.1129	0.000339995	Negative
gga-miR-206	17.67	0	4.23E-05	PLCXD1	27.0898	61.1129	0.000339995	Negative
gga-miR-218	2273.54	1690.07	1.58E-16	SAMD9L	15.1032	61.0749	0	Negative
gga-miR-106	65066.77	55680.53	5.82E-108	SAMD9L	15.1032	61.0749	0	Negative
gga-miR-221	154412.09	135297.34	1.07E-171	SAMD9L	15.1032	61.0749	0	Negative
gga-miR-20a	65325.93	59511.86	5.90E-30	SAMD9L	15.1032	61.0749	0	Negative
gga-miR-153	20691.55	18376.64	1.46E-18	CX3CR1	29.6454	57.9316	0.0233638	Negative
gga-miR-2964	136011.75	134807.91	1.66E-06	CX3CR1	29.6454	57.9316	0.0233638	Negative
gga-miR-30b	89639.82	73254.19	2.08E-267	IRF4	37.919	57.3401	0.0488699	Negative
gga-miR-30c	419880.02	323629.21	0	IRF4	37.919	57.3401	0.0488699	Negative
gga-miR-30d	2395549	2262888.5	1.05E-219	IRF4	37.919	57.3401	0.0488699	Negative
gga-miR-223	114460.26	97374.01	2.84E-206	IRF4	37.919	57.3401	0.0488699	Negative
gga-miR-1782	294.5	183.54	4.48E-06	IRF4	37.919	57.3401	0.0488699	Negative
gga-miR-125b	342385.36	306407.33	1.77E-244	IRF4	37.919	57.3401	0.0488699	Negative
gga-miR-153	20691.55	18376.64	1.46E-18	IRF4	37.919	57.3401	0.0488699	Negative
gga-miR-1744-3p	223.82	38.24	1.47E-31	SLBP	22.2286	55.0994	1.66E-05	Negative
gga-miR-1416-5p	1372.37	894.74	2.15E-20	SLBP	22.2286	55.0994	1.66E-05	Negative
gga-miR-196	241.49	130.01	8.20E-08	SLBP	22.2286	55.0994	1.66E-05	Negative
gga-miR-200a	541.88	53.53	6.11E-98	SLBP	22.2286	55.0994	1.66E-05	Negative
gga-miR-26a	6688359.39	6171874.21	0	IFIH1	25.6737	54.0603	0.000119819	Negative
gga-miR-2188	151484.76	138119.22	2.57E-66	IFIH1	25.6737	54.0603	0.000119819	Negative
gga-miR-34b	589	122.36	1.15E-70	IFIH1	25.6737	54.0603	0.000119819	Negative
gga-miR-29b	30498.39	26528.74	1.54E-39	COL6A2	27.5564	50.7635	0.000810331	Negative
gga-miR-125b	342385.36	306407.33	1.77E-244	TRANK1	22.5373	50.0981	9.02E-07	Negative
gga-miR-206	17.67	0	4.23E-05	MXRA8	27.842	48.3847	0.0191329	Negative
gga-miR-429	376.96	237.07	3.06E-07	FN1	16.9758	47.2428	4.30E-12	Negative
gga-miR-200b	388.74	53.53	4.43E-61	FN1	16.9758	47.2428	4.30E-12	Negative
gga-miR-153	20691.55	18376.64	1.46E-18	FN1	16.9758	47.2428	4.30E-12	Negative
gga-miR-128	107498.28	97029.88	1.06E-62	FN1	16.9758	47.2428	4.30E-12	Negative
gga-miR-27b	229915.92	214952.38	1.74E-38	FN1	16.9758	47.2428	4.30E-12	Negative
gga-miR-29b	30498.39	26528.74	1.54E-39	ARRDC2	25.5533	46.8829	0.0136235	Negative
gga-miR-24	30380.59	25641.64	5.81E-62	ARRDC2	25.5533	46.8829	0.0136235	Negative
gga-miR-449b-5p	58.9	7.65	1.83E-10	ARRDC2	25.5533	46.8829	0.0136235	Negative
gga-miR-449c-5p	106.02	30.59	3.00E-10	ARRDC2	25.5533	46.8829	0.0136235	Negative

gga-miR-34c	589	122.36	1.15E-70	ARRDC2	25.5533	46.8829	0.0136235	Negative
gga-miR-204	18812.64	12564.64	7.28E-237	HEBP1	24.5871	45.9593	0.00219329	Negative
gga-miR-200a	541.88	53.53	6.11E-98	HEBP1	24.5871	45.9593	0.00219329	Negative
gga-miR-211	18812.64	12564.64	7.28E-237	HEBP1	24.5871	45.9593	0.00219329	Negative
gga-miR-27b	229915.92	214952.38	1.74E-38	HEBP1	24.5871	45.9593	0.00219329	Negative
gga-miR-34b	589	122.36	1.15E-70	HEBP1	24.5871	45.9593	0.00219329	Negative
gga-miR-128	107498.28	97029.88	1.06E-62	HEBP1	24.5871	45.9593	0.00219329	Negative
gga-miR-200b	388.74	53.53	4.43E-61	RGL1	30.3201	45.761	0.0363964	Negative
gga-miR-429	376.96	237.07	3.06E-07	RGL1	30.3201	45.761	0.0363964	Negative
gga-miR-148a	1334325.18	1284575.26	1.74E-15	TXK	25.4733	44.9575	0.0454993	Negative
gga-miR-24	30380.59	25641.64	5.81E-62	TXK	25.4733	44.9575	0.0454993	Negative
gga-miR-221	154412.09	135297.34	1.07E-171	TXK	25.4733	44.9575	0.0454993	Negative
gga-miR-3531	29.45	0	6.47E-08	CTLA4	17.8859	44.5663	0.00578906	Negative
gga-miR-429	376.96	237.07	3.06E-07	CTLA4	17.8859	44.5663	0.00578906	Negative
gga-miR-200b	388.74	53.53	4.43E-61	CTLA4	17.8859	44.5663	0.00578906	Negative
gga-miR-200a	541.88	53.53	6.11E-98	IL18R1	22.0173	44.3116	0.00283805	Negative
gga-miR-106	65066.77	55680.53	5.82E-108	TSPO2	4.49306	42.8168	5.80E-12	Negative
gga-miR-20a	65325.93	59511.86	5.90E-30	TSPO2	4.49306	42.8168	5.80E-12	Negative
gga-miR-1744-3p	223.82	38.24	1.47E-31	ATP11B	0	42.341	1.68E-30	Negative
gga-miR-221	154412.09	135297.34	1.07E-171	ATP11B	0	42.341	1.68E-30	Negative
gga-miR-1782	294.5	183.54	4.48E-06	EIF2AK2	23.2727	42.1265	0.00496684	Negative
gga-miR-2964	136011.75	134807.91	1.66E-06	SNX10	25.6699	41.787	0.0348776	Negative
gga-miR-1416-5p	1372.37	894.74	2.15E-20	SNX10	25.6699	41.787	0.0348776	Negative
gga-miR-128	107498.28	97029.88	1.06E-62	SNX10	25.6699	41.787	0.0348776	Negative
gga-miR-30d	2395549	2262888.5	1.05E-219	SNX10	25.6699	41.787	0.0348776	Negative
gga-miR-30b	89639.82	73254.19	2.08E-267	SNX10	25.6699	41.787	0.0348776	Negative
gga-miR-30c	419880.02	323629.21	0	SNX10	25.6699	41.787	0.0348776	Negative
gga-miR-1655	217.93	91.77	6.84E-12	COL6A1	17.3967	40.0782	8.24E-07	Negative
gga-miR-429	376.96	237.07	3.06E-07	COL6A1	17.3967	40.0782	8.24E-07	Negative
gga-miR-200b	388.74	53.53	4.43E-61	COL6A1	17.3967	40.0782	8.24E-07	Negative
gga-miR-200a	541.88	53.53	6.11E-98	SOAT1	21.551	38.706	0.0301956	Negative
gga-miR-1416-5p	1372.37	894.74	2.15E-20	SOAT1	21.551	38.706	0.0301956	Negative
gga-miR-181b	154771.38	132460.16	5.52E-255	SOAT1	21.551	38.706	0.0301956	Negative
gga-miR-103	189492.89	186649.45	0.000170965	CHCHD3	0	38.0321	1.81E-07	Negative
gga-miR-15a	179756.73	178313.82	6.85E-09	CHCHD3	0	38.0321	1.81E-07	Negative
gga-miR-218	2273.54	1690.07	1.58E-16	CHCHD3	0	38.0321	1.81E-07	Negative
gga-miR-499	3145.26	1636.54	1.48E-97	CHCHD3	0	38.0321	1.81E-07	Negative
gga-miR-1744-3p	223.82	38.24	1.47E-31	CHCHD3	0	38.0321	1.81E-07	Negative
gga-miR-200a	541.88	53.53	6.11E-98	PLA2R1	18.8651	37.6376	8.34E-05	Negative
gga-miR-29b	30498.39	26528.74	1.54E-39	PLA2R1	18.8651	37.6376	8.34E-05	Negative
gga-miR-92	1955472.2	1805797.32	0	ARRDC3	18.2535	37.004	0.0176332	Negative
gga-miR-148a	1334325.18	1284575.26	1.74E-15	ARRDC3	18.2535	37.004	0.0176332	Negative
gga-miR-32	16079.68	13902.93	1.47E-23	ARRDC3	18.2535	37.004	0.0176332	Negative
gga-miR-365	1896.58	1460.65	6.00E-11	ARRDC3	18.2535	37.004	0.0176332	Negative
gga-miR-10b	9358782.65	8271177.74	0	ARRDC3	18.2535	37.004	0.0176332	Negative
gga-miR-33	6149.15	5070.21	1.15E-17	ABCA1	18.4027	36.868	3.26E-06	Negative
gga-miR-3531	29.45	0	6.47E-08	SLC7A5	18.1089	34.863	0.000280909	Negative
gga-miR-2964	136011.75	134807.91	1.66E-06	SGMS1	0	32.1293	2.75E-15	Negative
gga-miR-128	107498.28	97029.88	1.06E-62	SGMS1	0	32.1293	2.75E-15	Negative
gga-miR-27b	229915.92	214952.38	1.74E-38	SGMS1	0	32.1293	2.75E-15	Negative
gga-miR-429	376.96	237.07	3.06E-07	LAMA5	15.5551	30.0344	0.000119819	Negative
gga-miR-200b	388.74	53.53	4.43E-61	LAMA5	15.5551	30.0344	0.000119819	Negative
gga-miR-34c	589	122.36	1.15E-70	OCRL	0	28.6494	1.22E-20	Negative
gga-miR-449b-5p	58.9	7.65	1.83E-10	OCRL	0	28.6494	1.22E-20	Negative
gga-miR-128	107498.28	97029.88	1.06E-62	OCRL	0	28.6494	1.22E-20	Negative
gga-miR-449c-5p	106.02	30.59	3.00E-10	OCRL	0	28.6494	1.22E-20	Negative
gga-miR-30b	89639.82	73254.19	2.08E-267	OCRL	0	28.6494	1.22E-20	Negative
gga-miR-30c	419880.02	323629.21	0	OCRL	0	28.6494	1.22E-20	Negative
gga-miR-30d	2395549	2262888.5	1.05E-219	OCRL	0	28.6494	1.22E-20	Negative
gga-miR-214	43191.33	37778.03	2.13E-50	OCRL	0	28.6494	1.22E-20	Negative
gga-miR-125b	342385.36	306407.33	1.77E-244	TRANK1	8.58333	27.8523	0.00880009	Negative
gga-miR-218	2273.54	1690.07	1.58E-16	TNC	17.6498	27.1388	0.0213684	Negative
gga-miR-126-5p	734929.92	649284.98	0	TNC	17.6498	27.1388	0.0213684	Negative
gga-miR-1782	294.5	183.54	4.48E-06	TNC	17.6498	27.1388	0.0213684	Negative
gga-miR-128	107498.28	97029.88	1.06E-62	KIAA0319L	0	26.6076	1.46E-24	Negative
gga-miR-211	18812.64	12564.64	7.28E-237	KIAA0319L	0	26.6076	1.46E-24	Negative
gga-miR-204	18812.64	12564.64	7.28E-237	KIAA0319L	0	26.6076	1.46E-24	Negative
gga-miR-15a	179756.73	178313.82	6.85E-09	KIAA0319L	0	26.6076	1.46E-24	Negative
gga-miR-24	30380.59	25641.64	5.81E-62	KIF11	15.1507	26.21	0.0213877	Negative
gga-miR-27b	229915.92	214952.38	1.74E-38	SLC25A13	0	25.8201	3.49E-22	Negative
gga-miR-128	107498.28	97029.88	1.06E-62	SLC25A13	0	25.8201	3.49E-22	Negative
gga-miR-106	65066.77	55680.53	5.82E-108	SLC25A13	0	25.8201	3.49E-22	Negative
gga-miR-20a	65325.93	59511.86	5.90E-30	SLC25A13	0	25.8201	3.49E-22	Negative
gga-miR-27b	229915.92	214952.38	1.74E-38	PFKFB4	10.5021	25.4462	0.00663609	Negative
gga-miR-196	241.49	130.01	8.20E-08	PFKFB4	10.5021	25.4462	0.00663609	Negative
gga-miR-26a	6688359.39	6171874.21	0	PFKFB4	10.5021	25.4462	0.00663609	Negative
gga-miR-15a	179756.73	178313.82	6.85E-09	PFKFB4	10.5021	25.4462	0.00663609	Negative
gga-miR-200a	541.88	53.53	6.11E-98	TDH	7.78765	24.1951	0.000220576	Negative
gga-miR-30b	89639.82	73254.19	2.08E-267	TDH	7.78765	24.1951	0.000220576	Negative
gga-miR-30c	419880.02	323629.21	0	TDH	7.78765	24.1951	0.000220576	Negative
gga-miR-30d	2395549	2262888.5	1.05E-219	TDH	7.78765	24.1951	0.000220576	Negative
gga-miR-10a-5p	11492986.72[a]	10095802.89	0	TDH	7.78765	24.1951	0.000220576	Negative
gga-miR-10b	9358782.65	8271177.74	0	TDH	7.78765	24.1951	0.000220576	Negative
gga-miR-3525	465.31	298.25	4.12E-08	TDH	7.78765	24.1951	0.000220576	Negative
gga-miR-153	20691.55	18376.64	1.46E-18	RIF1	16.1349	24.1276	0.029507	Negative
gga-miR-23b	85858.45	79938	3.88E-18	PPP4R1L	0	22.214	3.57E-22	Negative
gga-miR-92	1955472.2	1805797.32	0	CNST	10.652	22.0176	0.0192507	Negative
gga-miR-32	16079.68	13902.93	1.47E-23	CNST	10.652	22.0176	0.0192507	Negative
gga-miR-2964	136011.75	134807.91	1.66E-06	TPH1	4.77094	18.5534	6.44E-05	Negative
gga-miR-20a	65325.93	59511.86	5.90E-30	TPH1	4.77094	18.5534	6.44E-05	Negative
gga-miR-106	65066.77	55680.53	5.82E-108	TPH1	4.77094	18.5534	6.44E-05	Negative
gga-miR-148a	1334325.18	1284575.26	1.74E-15	ATP8A1	9.6803	17.1941	0.040992	Negative
gga-miR-128	107498.28	97029.88	1.06E-62	ATP8A1	9.6803	17.1941	0.040992	Negative
gga-miR-137	94.24	45.88	0.000174111	GSTA3	3.96872	16.6754	0.00149743	Negative
gga-miR-15a	179756.73	178313.82	6.85E-09	GSTA3	3.96872	16.6754	0.00149743	Negative

gga-miR-128	107498.28	97029.88	1.06E-62	LOC769174	4.80644	15.2419	0.0335174	Negative
gga-miR-126-5p	734929.92	649284.98	0	SLC25A37	3.57351	14.2619	0.0020994	Negative
gga-miR-429	376.96	237.07	3.06E-07	IL1RL1	5.47556	13.7486	0.0196632	Negative
gga-miR-200b	388.74	53.53	4.43E-61	IL1RL1	5.47556	13.7486	0.0196632	Negative
gga-miR-23b	85858.45	79938	3.88E-18	IL1RL1	5.47556	13.7486	0.0196632	Negative
gga-miR-15a	179756.73	178313.82	6.85E-09	IL1RL1	5.47556	13.7486	0.0196632	Negative
gga-miR-148a	1334325.18	1284575.26	1.74E-15	ASPM	6.68208	12.042	0.0256369	Negative
gga-miR-223	114460.26	97374.01	2.84E-206	ASPM	6.68208	12.042	0.0256369	Negative
gga-miR-2964	136011.75	134807.91	1.66E-06	ASPM	6.68208	12.042	0.0256369	Negative
gga-miR-15a	179756.73	178313.82	6.85E-09	KANK1	4.85366	10.5986	0.010168	Negative
gga-miR-34b	589	122.36	1.15E-70	KANK1	4.85366	10.5986	0.010168	Negative
gga-miR-429	376.96	237.07	3.06E-07	KANK1	4.85366	10.5986	0.010168	Negative
gga-miR-1655	217.93	91.77	6.84E-12	KANK1	4.85366	10.5986	0.010168	Negative
gga-miR-26a	6688359.39	6171874.21	0	PDK4	1.95445	10.1462	0.000218755	Negative
gga-miR-449c-5p	106.02	30.59	3.00E-10	PDK4	1.95445	10.1462	0.000218755	Negative
gga-miR-429	376.96	237.07	3.06E-07	PDK4	1.95445	10.1462	0.000218755	Negative
gga-miR-200b	388.74	53.53	4.43E-61	PDK4	1.95445	10.1462	0.000218755	Negative
gga-miR-1416-5p	1372.37	894.74	2.15E-20	PDK4	1.95445	10.1462	0.000218755	Negative
gga-miR-34c	589	122.36	1.15E-70	PDK4	1.95445	10.1462	0.000218755	Negative
gga-miR-181b	154771.38	132460.16	5.52E-255	PDK4	1.95445	10.1462	0.000218755	Negative
gga-miR-449b-5p	58.9	7.65	1.83E-10	PDK4	1.95445	10.1462	0.000218755	Negative
gga-miR-383	1319.36	512.37	2.03E-76	RPS27L	6308.71	3976.31	0.0220731	Postive
gga-miR-200a	541.88	53.53	6.11E-98	RPS27L	6308.71	3976.31	0.0220731	Postive
gga-miR-1782	294.5	183.54	0.00000448	HSP90AA1	1016.75	616.806	0.0125173	Postive
gga-miR-181b	154771.38	132460.16	5.52E-255	HSP90AA1	1016.75	616.806	0.0125173	Postive
gga-miR-1655	217.93	91.77	6.84E-12	HSP90AA1	1016.75	616.806	0.0125173	Postive
gga-miR-20a	65325.93	59511.86	5.90E-30	CKB	200.702	139.093	0.0422742	Postive
gga-miR-106	65066.77	55680.53	5.82E-108	CKB	200.702	139.093	0.0422742	Postive
gga-miR-17-5p	90794.26	81719.84	4.22E-57	CKB	200.702	139.093	0.0422742	Postive
gga-miR-92	1955472.2	1805797.32	0	NAAA	269.254	139.021	5.44E-07	Postive
gga-miR-30b	89639.82	73254.19	2.08E-267	CALD1	157.1	109.112	0.0152297	Postive
gga-miR-30c	419880.02	323629.21	0	CALD1	157.1	109.112	0.0152297	Postive
gga-miR-148a	1334325.18	1284575.26	1.74E-15	MYH11	145.901	99.5936	0.000114137	Postive
gga-miR-218	2273.54	1690.07	1.58E-16	MYH11	145.901	99.5936	0.000114137	Postive
gga-miR-15a	179756.73	178313.82	6.85E-09	CSRP1	147.78	94.9176	0.0470582	Postive
gga-miR-27b	229915.92	214952.38	1.74E-38	SERPINH1	139.978	87.3562	0.0218924	Postive
gga-miR-214	43191.33	37778.03	2.13E-50	OLFML2B	92.1451	56.8475	0.0180725	Postive
gga-miR-211	18812.64	12564.64	7.28E-237	PTGS2	80.0275	51.021	0.027382	Postive
gga-miR-204	18812.64	12564.64	7.28E-237	PTGS2	80.0275	51.021	0.027382	Postive
gga-miR-32	16079.68	13902.93	1.47E-23	PTGS2	80.0275	51.021	0.027382	Postive
gga-miR-30b	89639.82	73254.19	2.08E-267	DPYSL2	69.437	45.1659	0.020673	Postive
gga-miR-30c	419880.02	323629.21	0	DPYSL2	69.437	45.1659	0.020673	Postive
gga-miR-30d	2395549	2262888.5	1.05E-219	DPYSL2	69.437	45.1659	0.020673	Postive
gga-miR-211	18812.64	12564.64	7.28E-237	RERGL	102.728	43.9204	0.00152603	Postive
gga-miR-204	18812.64	12564.64	7.28E-237	RERGL	102.728	43.9204	0.00152603	Postive
gga-miR-200b	388.74	53.53	4.43E-61	COL21A1	77.6023	40.2982	0.000154369	Postive
gga-miR-429	376.96	237.07	3.06E-07	COL21A1	77.6023	40.2982	0.000154369	Postive
gga-miR-1306	371.07	267.66	0.000422017	COL21A1	77.6023	40.2982	0.000154369	Postive
gga-miR-1729-5p	3687.14	3280.72	0.000504673	COL21A1	77.6023	40.2982	0.000154369	Postive
gga-miR-92	1955472.2	1805797.32	0	COL1A2	60.9794	37.0589	0.000583141	Postive
gga-miR-32	16079.68	13902.93	1.47E-23	COL1A2	60.9794	37.0589	0.000583141	Postive
gga-let-7b	219225.59	155761.71	0	COL1A2	60.9794	37.0589	0.000583141	Postive
gga-let-7c	1206765.58	888120.11	0	COL1A2	60.9794	37.0589	0.000583141	Postive
gga-let-7f	2489453.18	2100290.04	0	COL1A2	60.9794	37.0589	0.000583141	Postive
gga-let-7j	2472908.19	2066848.07	0	COL1A2	60.9794	37.0589	0.000583141	Postive
gga-let-7g	415491.97	344797.14	0	COL1A2	60.9794	37.0589	0.000583141	Postive
gga-miR-26a	6688359.39	6171874.21	0	COL1A2	60.9794	37.0589	0.000583141	Postive
gga-miR-206	17.67	0	4.23E-05	THBS1	71.0634	36.227	7.06E-05	Postive
gga-miR-32	16079.68	13902.93	1.47E-23	NGFR	92.3428	30.1858	0.000261887	Postive
gga-miR-92	1955472.2	1805797.32	0	NGFR	92.3428	30.1858	0.000261887	Postive
gga-miR-137	94.24	45.88	0.000174111	CNRIP1	56.1778	29.0769	0.0451103	Postive
gga-miR-200a	541.88	53.53	6.11E-98	CNRIP1	56.1778	29.0769	0.0451103	Postive
gga-miR-429	376.96	237.07	3.06E-07	ATP1B4	49.2532	24.085	0.00184808	Postive
gga-miR-200b	388.74	53.53	4.43E-61	ATP1B4	49.2532	24.085	0.00184808	Postive
gga-miR-153	20691.55	18376.64	1.46E-18	EDN3	51.7529	21.1925	0.00273177	Postive
gga-miR-1744-3p	223.82	38.24	1.47E-31	EDN3	51.7529	21.1925	0.00273177	Postive
gga-miR-27b	229915.92	214952.38	1.74E-38	LAMA4	31.0764	18.3277	0.0072436	Postive
gga-miR-499	3145.26	1636.54	1.48E-97	LAMA4	31.0764	18.3277	0.0072436	Postive
gga-miR-30d	2395549	2262888.5	1.05E-219	LAMA4	31.0764	18.3277	0.0072436	Postive
gga-miR-30b	89639.82	73254.19	2.08E-267	LAMA4	31.0764	18.3277	0.0072436	Postive
gga-miR-30c	419880.02	323629.21	0	LAMA4	31.0764	18.3277	0.0072436	Postive
gga-miR-206	17.67	0	4.23E-05	LAMA4	31.0764	18.3277	0.0072436	Postive
gga-miR-211	18812.64	12564.64	7.28E-237	LAMA4	31.0764	18.3277	0.0072436	Postive
gga-miR-218	2273.54	1690.07	1.58E-16	DAB1	28.7618	16.1637	0.0087455	Postive
gga-miR-15a	179756.73	178313.82	6.85E-09	DAB1	28.7618	16.1637	0.0087455	Postive
gga-miR-221	154412.09	135297.34	1.07E-171	DAB1	28.7618	16.1637	0.0087455	Postive
gga-miR-26a	6688359.39	6171874.21	0	COL22A1	33.0758	14.9105	5.32E-06	Postive
gga-miR-126-5p	734929.92	649284.98	0	COL22A1	33.0758	14.9105	5.32E-06	Postive
gga-miR-15a	179756.73	178313.82	6.85E-09	PKDCC	32.4038	14.8473	0.0140127	Postive
gga-miR-2130	29.45	7.65	0.000695053	PKDCC	32.4038	14.8473	0.0140127	Postive
gga-miR-2964	136011.75	134807.91	1.66E-06	PKDCC	32.4038	14.8473	0.0140127	Postive
gga-miR-92	1955472.2	1805797.32	0	NPNT	25.4981	10.4204	0.00199284	Postive
gga-miR-32	16079.68	13902.93	1.47E-23	NPNT	25.4981	10.4204	0.00199284	Postive
gga-miR-29b	30498.39	26528.74	1.54E-39	ZDHHC23	21.5787	9.26066	9.77E-06	Postive
gga-miR-126-5p	734929.92	649284.98	0	ZDHHC23	21.5787	9.26066	9.77E-06	Postive
gga-miR-148a	1334325.18	1284575.26	1.74E-15	ZDHHC23	21.5787	9.26066	9.77E-06	Postive
gga-miR-200b	388.74	53.53	4.43E-61	PTPN13	16.9784	8.02404	1.98E-05	Postive
gga-miR-429	376.96	237.07	3.06E-07	PTPN13	16.9784	8.02404	1.98E-05	Postive
gga-miR-23b	85858.45	79938	3.88E-18	TTC7B	14.887	7.66349	0.0265443	Postive
gga-miR-27b	229915.92	214952.38	1.74E-38	TTC7B	14.887	7.66349	0.0265443	Postive
gga-miR-206	17.67	0	4.23E-05	TTC7B	14.887	7.66349	0.0265443	Postive
gga-miR-148a	1334325.18	1284575.26	1.74E-15	RXFP1	17.4894	6.82476	0.000914608	Postive
gga-miR-23b	85858.45	79938	3.88E-18	GPR64	18.6512	6.77161	9.47E-05	Postive
gga-miR-2964	136011.75	134807.91	1.66E-06	GPR64	18.6512	6.77161	9.47E-05	Postive
gga-miR-449c-5p	106.02	30.59	3.00E-10	GPR64	18.6512	6.77161	9.47E-05	Postive

gga-miR-34c	589	122.36	1.15E-70	GPR64	18.6512	6.77161	9.47E-05	Postive
gga-miR-196	241.49	130.01	8.20E-08	ANO1	14.7669	6.73814	0.00836706	Postive
gga-miR-200a	541.88	53.53	6.11E-98	GPM6B	16.9083	6.64639	0.0143089	Postive
gga-miR-34c	589	122.36	1.15E-70	MYOF	13.0386	6.61211	0.00106217	Postive
gga-miR-449b-5p	58.9	7.65	1.83E-10	MYOF	13.0386	6.61211	0.00106217	Postive
gga-miR-20a	65325.93	59511.86	5.90E-30	MYOF	13.0386	6.61211	0.00106217	Postive
gga-miR-106	65066.77	55680.53	5.82E-108	MYOF	13.0386	6.61211	0.00106217	Postive
gga-miR-92	1955472.2	1805797.32	0	MYOF	13.0386	6.61211	0.00106217	Postive
gga-miR-1744-3p	223.82	38.24	1.47E-31	CCDC48	14.7045	5.57979	0.0251733	Postive
gga-miR-218	2273.54	1690.07	1.58E-16	CTNNA2	13.1785	4.97915	0.00190025	Postive
gga-miR-181b	154771.38	132460.16	5.52E-255	TSPAN8	13.7763	3.33289	0.00136607	Postive
gga-miR-181a-5p	3651195.65	3273091.29	0	TSPAN8	13.7763	3.33289	0.00136607	Postive
gga-miR-23b	85858.45	79938	3.88E-18	TSPAN8	13.7763	3.33289	0.00136607	Postive
gga-miR-103	189492.89	186649.45	0.000170965	SEMA3E	13.1018	3.00007	7.69E-07	Postive
gga-miR-499	3145.26	1636.54	1.48E-97	SEMA3E	13.1018	3.00007	7.69E-07	Postive
gga-miR-218	2273.54	1690.07	1.58E-16	SEMA3E	13.1018	3.00007	7.69E-07	Postive
gga-miR-153	20691.55	18376.64	1.46E-18	ROCK1	69.1777	0	2.67E-24	Postive
gga-miR-33	6149.15	5070.21	1.15E-17	ROCK1	69.1777	0	2.67E-24	Postive
gga-miR-449b-5p	58.9	7.65	1.83E-10	MORC2	33.9424	0	5.32E-23	Postive
gga-miR-449c-5p	106.02	30.59	3.00E-10	MORC2	33.9424	0	5.32E-23	Postive
gga-miR-34c	589	122.36	1.15E-70	MORC2	33.9424	0	5.32E-23	Postive
gga-miR-429	376.96	237.07	3.06E-07	HEATR3	22.1763	0	2.63E-16	Postive
gga-miR-15b	159029.84	161443.71	1.32E-33	ANG	444.106	995.964	1.82E-10	Postive
gga-miR-146a	290082.22	342862.36	0	STAT1	204.763	424.888	3.26E-06	Postive
gga-miR-146c-5p	1645422.9	2124455.74	0	STAT1	204.763	424.888	3.26E-06	Postive
gga-miR-1458	29.45	68.83	8.01E-05	CA2	62.5318	314.61	0	Postive
gga-miR-454-3p	25474.23	26024.01	6.52E-08	IRF1	187.675	283.533	0.0194019	Postive
gga-miR-194	176.7	412.96	9.89E-24	SAMD13	200.057	278.083	0.0451103	Postive
gga-miR-1329-5p	371.07	619.44	2.15E-16	SAMD13	200.057	278.083	0.0451103	Postive
gga-miR-1563	624.34	963.57	3.80E-19	SAMD13	200.057	278.083	0.0451103	Postive
gga-miR-144	33785.01	56537.03	0	SAMD13	200.057	278.083	0.0451103	Postive
gga-miR-1699	0	15.29	0.000124084	BIRC2	147.57	247.753	8.34E-05	Postive
gga-miR-199-5p	148180.47	169687.58	0	CXCR4	133.009	243.903	0.000633073	Postive
gga-miR-20b	20880.03	23599.8	7.61E-56	CXCR4	133.009	243.903	0.000633073	Postive
gga-miR-1756a	259.16	382.37	5.07E-07	CXCR4	133.009	243.903	0.000633073	Postive
gga-miR-194	176.7	412.96	9.89E-24	CXCR4	133.009	243.903	0.000633073	Postive
gga-miR-1626-5p	0	15.29	0.000124084	PLBD1	86.9448	153.767	0.00199253	Postive
gga-miR-19b	246119.3	249258.5	5.42E-46	LIPA	95.946	152.17	0.0268453	Postive
gga-miR-19a	234463	235546.76	1.63E-28	LIPA	95.946	152.17	0.0268453	Postive
gga-miR-1452	0	22.94	1.53E-06	LIPA	95.946	152.17	0.0268453	Postive
gga-miR-454-3p	25474.23	26024.01	6.52E-08	LIPA	95.946	152.17	0.0268453	Postive
gga-miR-138	530.1	650.03	0.000170237	LOC417345	78.4619	133.996	0.0115828	Postive
gga-miR-30e	2745756.28	2781204.55	0	TXNDC5	79.2419	128.407	0.0159887	Postive
gga-miR-1458	29.45	68.83	8.01E-05	TXNDC5	79.2419	128.407	0.0159887	Postive
gga-miR-1699	0	15.29	0.000124084	AK2	63.2963	108.723	0.0141272	Postive
gga-miR-19a	234463	235546.76	1.63E-28	GBP7	66.3665	107.194	0.0180635	Postive
gga-miR-19b	246119.3	249258.5	5.42E-46	GBP7	66.3665	107.194	0.0180635	Postive
gga-miR-9-3p	117.8	565.91	9.06E-73	BNIP3	59.8149	105.039	0.0103493	Postive
gga-miR-15b	159029.84	161443.71	1.32E-33	BNIP3	59.8149	105.039	0.0103493	Postive
gga-miR-16	229821.69	230208.89	2.43E-23	BNIP3	59.8149	105.039	0.0103493	Postive
gga-miR-1626-5p	0	15.29	0.000124084	CD4	55.0231	98.3413	0.00103327	Postive
gga-miR-194	176.7	412.96	9.89E-24	RGS1	52.0715	96.0099	0.00837528	Postive
gga-miR-21	2604101.92	4456262.37	0	RGS1	52.0715	96.0099	0.00837528	Postive
gga-miR-15b	159029.84	161443.71	1.32E-33	NMI	56.2541	94.0707	0.0215854	Postive
gga-miR-142-5p	3575096.92	3622805.69	0	FAS	60.1003	92.4175	0.0276367	Postive
gga-miR-490	907.06	1124.16	1.54E-07	SLC43A2	41.6474	91.733	6.60E-06	Postive
gga-miR-18a	9088.26	9444.51	1.63E-05	SLC43A2	41.6474	91.733	6.60E-06	Postive
gga-miR-18b	8446.25	8832.72	4.71E-06	SLC43A2	41.6474	91.733	6.60E-06	Postive
gga-miR-20b	20880.03	23599.8	7.61E-56	KPNA2	51.9748	90.9272	0.00403041	Postive
gga-miR-1769-3p	965.96	1537.12	2.70E-33	LYG2	12.4333	81.3108	8.63E-12	Postive
gga-miR-490	907.06	1124.16	1.54E-07	SLC6A6	50.6603	78.9541	0.0442082	Postive
gga-miR-454-3p	25474.23	26024.01	6.52E-08	SLC6A6	50.6603	78.9541	0.0442082	Postive
gga-miR-130c-3p	99411.32	101679.48	3.40E-29	SLC6A6	50.6603	78.9541	0.0442082	Postive
gga-miR-130a	91129.99	93596.21	2.70E-31	SLC6A6	50.6603	78.9541	0.0442082	Postive
gga-miR-19b	246119.3	249258.5	5.42E-46	SLC6A6	50.6603	78.9541	0.0442082	Postive
gga-miR-19a	234463	235546.76	1.63E-28	SLC6A6	50.6603	78.9541	0.0442082	Postive
gga-miR-18a	9088.26	9444.51	1.63E-05	SLC6A6	50.6603	78.9541	0.0442082	Postive
gga-miR-18b	8446.25	8832.72	4.71E-06	SLC6A6	50.6603	78.9541	0.0442082	Postive
gga-miR-155	121828.64	168379.88	0	MX1	9.4955	74.9547	0	Postive
gga-miR-34a	3115.81	3785.45	1.13E-19	RNF213	30.988	70.4357	1.51E-05	Postive
gga-miR-3536	58.9	152.95	4.53E-11	NFE2L2	40.7554	63.9418	0.0194306	Postive
gga-miR-1563	624.34	963.57	3.80E-19	ZCCHC11	36.7354	62.2242	0.00521562	Postive
gga-miR-144	33785.01	56537.03	0	ZCCHC11	36.7354	62.2242	0.00521562	Postive
gga-miR-30e	2745756.28	2781204.55	0	CFH	40.0538	61.5802	0.0449451	Postive
gga-miR-20b	20880.03	23599.8	7.61E-56	PLCXD1	27.0898	61.1129	0.000339995	Postive
gga-miR-133b	15514.24	16694.22	3.94E-19	PLCXD1	27.0898	61.1129	0.000339995	Postive
gga-miR-133a	17092.76	18384.29	9.06E-21	PLCXD1	27.0898	61.1129	0.000339995	Postive
gga-miR-133c	15690.94	16984.82	1.41E-21	PLCXD1	27.0898	61.1129	0.000339995	Postive
gga-miR-155	121828.64	168379.88	0	CX3CR1	29.6454	57.9316	0.0233638	Postive
gga-miR-30e	2745756.28	2781204.55	0	IRF4	37.919	57.3401	0.0488699	Postive
gga-miR-144	33785.01	56537.03	0	IRF4	37.919	57.3401	0.0488699	Postive
gga-miR-194	176.7	412.96	9.89E-24	IRF4	37.919	57.3401	0.0488699	Postive
gga-miR-199-5p	148180.47	169687.58	0	IRF4	37.919	57.3401	0.0488699	Postive
gga-miR-1662	2621.05	3655.44	5.93E-45	SLBP	22.2286	55.0994	1.66E-05	Postive
gga-miR-1712-3p	23.56	61.18	4.80E-05	SLBP	22.2286	55.0994	1.66E-05	Postive
gga-miR-146a	290082.22	342862.36	0	ZC3HAV1	30.5611	51.9723	0.00521562	Postive
gga-miR-1563	624.34	963.57	3.80E-19	ZC3HAV1	30.5611	51.9723	0.00521562	Postive
gga-miR-1712-3p	23.56	61.18	4.80E-05	ZC3HAV1	30.5611	51.9723	0.00521562	Postive
gga-miR-146c-5p	1645422.9	2124455.74	0	ZC3HAV1	30.5611	51.9723	0.00521562	Postive
gga-miR-1769-3p	965.96	1537.12	2.70E-33	CX3CL1	20.8852	51.5959	0.0025114	Postive
gga-miR-183	4252.58	4619.01	5.56E-07	NRP2	33.5538	51.5064	0.0143089	Postive
gga-miR-19b	246119.3	249258.5	5.42E-46	NRP2	33.5538	51.5064	0.0143089	Postive
gga-miR-19a	234463	235546.76	1.63E-28	NRP2	33.5538	51.5064	0.0143089	Postive
gga-miR-1668-3p	53.01	114.71	1.65E-06	COL6A2	27.5564	50.7635	0.000810331	Postive
gga-miR-1329-5p	371.07	619.44	2.15E-16	FN1	16.9758	47.2428	4.30E-12	Postive

gga-miR-144	33785.01	56537.03	0	FN1	16.9758	47.2428	4.30E-12	Postive
gga-miR-1585	0	15.29	0.000124084	ARRDC2	25.5533	46.8829	0.0136235	Postive
gga-miR-34a	3115.81	3785.45	1.13E-19	ARRDC2	25.5533	46.8829	0.0136235	Postive
gga-miR-1699	0	15.29	0.000124084	HEBP1	24.5871	45.9593	0.00219329	Postive
gga-miR-146c-5p	1645422.9	2124455.74	0	HEBP1	24.5871	45.9593	0.00219329	Postive
gga-miR-146a	290082.22	342862.36	0	HEBP1	24.5871	45.9593	0.00219329	Postive
gga-miR-19a	234463	235546.76	1.63E-28	RGL1	30.3201	45.761	0.0363964	Postive
gga-miR-19b	246119.3	249258.5	5.42E-46	RGL1	30.3201	45.761	0.0363964	Postive
gga-miR-155	121828.64	168379.88	0	RGL1	30.3201	45.761	0.0363964	Postive
gga-miR-21	2604101.92	4456262.37	0	TXK	25.4733	44.9575	0.0454993	Postive
gga-miR-3535	0	30.59	2.24E-08	TXK	25.4733	44.9575	0.0454993	Postive
gga-miR-155	121828.64	168379.88	0	CTLA4	17.8859	44.5663	0.00578906	Postive
gga-miR-138	530.1	650.03	0.000170237	TPX2	25.8939	44.2807	0.0159887	Postive
gga-miR-1587	0	15.29	0.000124084	TPX2	25.8939	44.2807	0.0159887	Postive
gga-miR-199-5p	148180.47	169687.58	0	SNTB1	23.4159	43.1244	7.60E-05	Postive
gga-miR-1756a	259.16	382.37	5.07E-07	SNTB1	23.4159	43.1244	7.60E-05	Postive
gga-miR-20b	20880.03	23599.8	7.61E-56	TSPO2	4.49306	42.8168	5.80E-12	Postive
gga-miR-3535	0	30.59	2.24E-08	SNX10	25.6699	41.787	0.0348776	Postive
gga-miR-30e	2745756.28	2781204.55	0	SNX10	25.6699	41.787	0.0348776	Postive
gga-miR-190	1666.87	1934.79	3.39E-07	SNX10	25.6699	41.787	0.0348776	Postive
gga-miR-144	33785.01	56537.03	0	SOAT1	21.551	38.706	0.0301956	Postive
gga-miR-146a	290082.22	342862.36	0	TNFRSF18	22.0424	38.1007	0.0156132	Postive
gga-miR-146c-5p	1645422.9	2124455.74	0	TNFRSF18	22.0424	38.1007	0.0156132	Postive
gga-miR-1699	0	15.29	0.000124084	TNFRSF18	22.0424	38.1007	0.0156132	Postive
gga-miR-15b	159029.84	161443.71	1.32E-33	CHCHD3	0	38.0321	1.81E-07	Postive
gga-miR-16c	28083.49	29151.79	1.86E-14	CHCHD3	0	38.0321	1.81E-07	Postive
gga-miR-16	229821.69	230208.89	2.43E-23	CHCHD3	0	38.0321	1.81E-07	Postive
gga-miR-15c-5p	223631.3	224458.07	8.92E-26	CHCHD3	0	38.0321	1.81E-07	Postive
gga-miR-155	121828.64	168379.88	0	PLA2R1	18.8651	37.6376	8.34E-05	Postive
gga-miR-155	121828.64	168379.88	0	ARRDC3	18.2535	37.004	0.0176332	Postive
gga-miR-19b	246119.3	249258.5	5.42E-46	ARRDC3	18.2535	37.004	0.0176332	Postive
gga-miR-144	33785.01	56537.03	0	ARRDC3	18.2535	37.004	0.0176332	Postive
gga-miR-130a	91129.99	93596.21	2.70E-31	ARRDC3	18.2535	37.004	0.0176332	Postive
gga-miR-130c-3p	99411.32	101679.48	3.40E-29	ARRDC3	18.2535	37.004	0.0176332	Postive
gga-miR-199-5p	148180.47	169687.58	0	SLC7A5	18.1089	34.863	0.000280909	Postive
gga-miR-147	9553.57	16342.44	0	SLC7A5	18.1089	34.863	0.000280909	Postive
gga-miR-1458	29.45	68.83	8.01E-05	SGMS1	0	32.1293	2.75E-15	Postive
gga-miR-3535	0	30.59	2.24E-08	SGMS1	0	32.1293	2.75E-15	Postive
gga-miR-144	33785.01	56537.03	0	SGMS1	0	32.1293	2.75E-15	Postive
gga-miR-1769-3p	965.96	1537.12	2.70E-33	SGMS1	0	32.1293	2.75E-15	Postive
gga-miR-9-3p	117.8	565.91	9.06E-73	GZMA	15.9436	31.8832	0.0340161	Postive
gga-miR-1756a	259.16	382.37	5.07E-07	LAMA5	15.5551	30.0344	0.000119819	Postive
gga-miR-142-3p	470663.55	571113.52	0	IL6ST	17.0215	29.8613	0.0273944	Postive
gga-miR-34a	3115.81	3785.45	1.13E-19	OCRL	0	28.6494	1.22E-20	Postive
gga-miR-21	2604101.92	4456262.37	0	OCRL	0	28.6494	1.22E-20	Postive
gga-miR-30e	2745756.28	2781204.55	0	OCRL	0	28.6494	1.22E-20	Postive
gga-miR-19b	246119.3	249258.5	5.42E-46	OCRL	0	28.6494	1.22E-20	Postive
gga-miR-19a	234463	235546.76	1.63E-28	OCRL	0	28.6494	1.22E-20	Postive
gga-miR-130c-3p	99411.32	101679.48	3.40E-29	OCRL	0	28.6494	1.22E-20	Postive
gga-miR-21	2604101.92	4456262.37	0	TNC	17.6498	27.1388	0.0213684	Postive
gga-miR-16	229821.69	230208.89	2.43E-23	KIAA0319L	0	26.6076	1.46E-24	Postive
gga-miR-16c	28083.49	29151.79	1.86E-14	KIAA0319L	0	26.6076	1.46E-24	Postive
gga-miR-15b	159029.84	161443.71	1.32E-33	KIAA0319L	0	26.6076	1.46E-24	Postive
gga-miR-1329-5p	371.07	619.44	2.15E-16	KIF11	15.1507	26.21	0.0213877	Postive
gga-miR-146c-5p	1645422.9	2124455.74	0	KIF11	15.1507	26.21	0.0213877	Postive
gga-miR-146a	290082.22	342862.36	0	KIF11	15.1507	26.21	0.0213877	Postive
gga-miR-1329-5p	371.07	619.44	2.15E-16	SLC25A13	0	25.8201	3.49E-22	Postive
gga-miR-133c	15690.94	16984.82	1.41E-21	SLC25A13	0	25.8201	3.49E-22	Postive
gga-miR-133a	17092.76	18384.29	9.06E-21	SLC25A13	0	25.8201	3.49E-22	Postive
gga-miR-133b	15514.24	16694.22	3.94E-19	SLC25A13	0	25.8201	3.49E-22	Postive
gga-miR-20b	20880.03	23599.8	7.61E-56	SLC25A13	0	25.8201	3.49E-22	Postive
gga-miR-16	229821.69	230208.89	2.43E-23	PFKFB4	10.5021	25.4462	0.00663609	Postive
gga-miR-15b	159029.84	161443.71	1.32E-33	PFKFB4	10.5021	25.4462	0.00663609	Postive
gga-miR-144	33785.01	56537.03	0	TDH	7.78765	24.1951	0.000220576	Postive
gga-miR-30e	2745756.28	2781204.55	0	TDH	7.78765	24.1951	0.000220576	Postive
gga-miR-1756a	259.16	382.37	5.07E-07	FAM82A2	0	20.277	2.38E-11	Postive
gga-miR-3536	58.9	152.95	4.53E-11	TPH1	4.77094	18.5534	6.44E-05	Postive
gga-miR-20b	20880.03	23599.8	7.61E-56	TPH1	4.77094	18.5534	6.44E-05	Postive
gga-miR-1563	624.34	963.57	3.80E-19	GSTA3	3.96872	16.6754	0.00149743	Postive
gga-miR-16c	28083.49	29151.79	1.86E-14	GSTA3	3.96872	16.6754	0.00149743	Postive
gga-miR-16	229821.69	230208.89	2.43E-23	GSTA3	3.96872	16.6754	0.00149743	Postive
gga-miR-15b	159029.84	161443.71	1.32E-33	GSTA3	3.96872	16.6754	0.00149743	Postive
gga-miR-21	2604101.92	4456262.37	0	GSTA3	3.96872	16.6754	0.00149743	Postive
gga-miR-1674	259.16	512.37	4.29E-21	SLC25A37	3.57351	14.2619	0.0020994	Postive
gga-miR-1668-3p	53.01	114.71	1.65E-06	SLC25A37	3.57351	14.2619	0.0020994	Postive
gga-miR-1563	624.34	963.57	3.80E-19	IL1RL1	5.47556	13.7486	0.0196632	Postive
gga-miR-15b	159029.84	161443.71	1.32E-33	IL1RL1	5.47556	13.7486	0.0196632	Postive
gga-miR-16c	28083.49	29151.79	1.86E-14	IL1RL1	5.47556	13.7486	0.0196632	Postive
gga-miR-16	229821.69	230208.89	2.43E-23	IL1RL1	5.47556	13.7486	0.0196632	Postive
gga-miR-1458	29.45	68.83	8.01E-05	IL1RL1	5.47556	13.7486	0.0196632	Postive
gga-miR-3536	58.9	152.95	4.53E-11	ASPM	6.68208	12.042	0.0256369	Postive
gga-miR-22-3p	2389181.92	3014197.09	0	ASPM	6.68208	12.042	0.0256369	Postive
gga-miR-144	33785.01	56537.03	0	ASPM	6.68208	12.042	0.0256369	Postive
gga-miR-130c-3p	99411.32	101679.48	3.40E-29	ASPM	6.68208	12.042	0.0256369	Postive
gga-miR-130a	91129.99	93596.21	2.70E-31	ASPM	6.68208	12.042	0.0256369	Postive
gga-miR-454-3p	25474.23	26024.01	6.52E-08	ASPM	6.68208	12.042	0.0256369	Postive
gga-miR-15b	159029.84	161443.71	1.32E-33	KANK1	4.85366	10.5986	0.010168	Postive
gga-miR-16	229821.69	230208.89	2.43E-23	KANK1	4.85366	10.5986	0.010168	Postive
gga-miR-16c	28083.49	29151.79	1.86E-14	KANK1	4.85366	10.5986	0.010168	Postive
gga-miR-34a	3115.81	3785.45	1.13E-19	PDK4	1.95445	10.1462	0.000218755	Postive

[a]The correlation expression patterns between miRNAs and their targets.

Table S4. Gene ontology (GO) funciton annotation of the 111 genes was also performed by STRING.

GO_id	Go Term	Number of genes	p value	Type	Differentially expressed genes
GO:0006950	response to stress	24	2.78E-06	biological_process	IRF1; SGMS1; RSFR; ABCA1; TXNDC5; IRF4; BNIP3; ABCC9; THBS1; PTGS2; TNC; FN1; PLA2R;PDK4; MDK; CXCR4; LIPA; C7; IFIH1; EIF2AK2; NFE2L2; CTLA4; HSP90AA1; RCSD1
GO:0006952	defense response	11	3.11E-05	biological_process	IRF1; SGMS1; IRF4; BNIP3; ABCC9; THBS1; FN1; MDK; LIPA; C7; IFIH1
GO:0045589	regulation of regulatory T cell differentiation	2	6.27E-05	biological_process	IRF1; CTLA4
GO:0045590	negative regulation of regulatory T cell differentiation	2	6.27E-05	biological_process	IRF1; CTLA4
GO:0046632	alpha-beta T cell differentiation	4	6.62E-05	biological_process	IRF1; IRF4; TXK; LOC429317
GO:0046631	alpha-beta T cell activation	4	8.76E-05	biological_process	IRF1; IRF4; TXK; LOC429317
GO:0002252	immune effector process	7	1.26E-04	biological_process	IRF1; IRF4;BNIP3; ABCC9; C7; CX3CR1; LOC429317
GO:0032369	negative regulation of lipid transport	3	1.32E-04	biological_process	SREBF2; THBS1; PLA2RA
GO:0051239	regulation of multicellular organismal process	21	1.59E-04	biological_process	IRF1; LOC429317; NGFR; ANKRD15; LAMA4; IRF4; PTGS2; THBS1; DAB1; CXCR4; PDK4; SEMA3E; IL1RL1; EIF2AK2; NFE2L2; TXK; CX3CR1; CTLA4; ARRDC3; EDN3; LGMN
GO:0048583	regulation of response to stimulus	26	1.79E-04	biological_process	IRF1; SGMS1; ANKRD15; ABCA; BNIP3; IRF4; LOC429317; NGFR; PTGS2; THBS1; DAB1; CXCR4; MDK; RGS1; IL1RL1; PLA2R1; C7; RGL1; CX3CR1; EIF2AK2; NFE2L2; CTLA4; TXK; ARRDC3; EDN3; OCRL
GO:0032368	regulation of lipid transport	4	3.64E-04	biological_process	SREBF2; THBS1; PLA2RA; ABCA1
GO:0050873	brown fat cell differentiation	3	3.65E-04	biological_process	LAMA4; BNIP3; PTGS2
GO:0045332	phospholipid translocation	3	4.31E-04	biological_process	ATP11B; ABCA1; ATP8A1
GO:0009615	response to virus	5	5.35E-04	biological_process	IRF1; BNIP3; EIF2AK2; IFIH1; ABCC9
GO:0051240	positive regulation of multicellular organismal process	9	5.52E-04	biological_process	IRF1; LOC429317; PTGS2; THBS1; IL1RL1; EIF2AK2; NFE2L2; TXK; EDN3
GO:0034377	plasma lipoprotein particle assembly	2	6.17E-04	biological_process	ABCA1; SOAT1
GO:2000192	negative regulation of fatty acid transport	2	6.17E-04	biological_process	THBS1; PLA2R1
GO:0034616	response to laminar fluid shear stress	2	6.17E-04	biological_process	ABCA1; NFE2L2
GO:0032891	negative regulation of organic acid transport	2	6.17E-04	biological_process	THBS1; PLA2R1
GO:0061024	membrane organization	8	6.43E-04	biological_process	CXCR4; CHCHD3; ATP11B; ABCA1; BNIP3; THBS1; ZDHHC23; ATP8A1
GO:0051093	negative regulation of developmental process	10	7.83E-04	biological_process	IRF1; RIF1; SEMA3E; DAB1; CX3CR1; CTLA4; NGIR; ANKRD15; BNIP3; THBS1
GO:0050795	regulation of behavior	5	8.11E-04	biological_process	EDN3; ARRDC3; CXCR4; MDK; THBS1
GO:0043032	positive regulation of macrophage activation	2	9.21E-04	biological_process	THBS1; IL1RL1
GO:0055098	response to low-density lipoprotein particle	2	9.21E-04	biological_process	SREBF2; ABCA1
GO:0031650	regulation of heat generation	2	9.21E-04	biological_process	PTGS2; ARRDC3
GO:0097035	regulation of membrane lipid distribution	3	9.88E-04	biological_process	ATP11B; ABCA1; ATP8A1
GO:0010812	negative regulation of cell-substrate adhesion	3	9.88E-04	biological_process	ANKRD15; THBS1; SEMA3E

GO:0001816	cytokine production	4	1.00E-03	biological_process	ABCA1; PLA2R1; TXK; LIPA
GO:0005604	basement membrane	5	0.0000693	cellular_component	RSFR; LAMA4; TNC; FN1; MDK
GO:0044420	extracellular matrix part	5	0.000262	cellular_component	RSFR; LAMA4; TNC; FN1; MDK
GO:0042383	sarcolemma	4	0.000279	cellular_component	COL6A1; COL6A2; ABCC9; LAMP1
GO:0005578	proteinaceous extracellular matrix	7	0.000583	cellular_component	RSFR; LAMA4; COL6A1; COL6A2; TNC; FN1; MDK
GO:0005577	fibrinogen complex	2	0.000978	cellular_component	THBS1; FN1
GO:0008281	sulfonylurea receptor activity	2	0.0000607	molecular_function	ABCC8; ABCC9
GO:0005548	phospholipid transporter activity	3	0.000833	molecular_function	ABCA1; ATP11B; ATP8A1
GO:0016894	endonuclease activity, active with either ribo-or deoxyribonucleic acids and producing 3'−phosphomonoesters	2	0.000892	molecular_function	RSFR; DNASE2B

Table S5. Primer pairs employed to identify ALVs, MDV and REV.

Virus be specific detected	Primer pairs	Primer sequence (5' to 3')	Size of PCR product	Reference
Subgroup J ALV	H5	GGATGAGGTGACTAAGAAAG	545 bp	Smith *et al* ., 1998
	H7	CGAACCAAAGGTAACACACG		
Subgroups A-E ALV	H5	GGATGAGGTGACTAAGAAAG	295-326[a] bp	Smith *et al* ., 1998
	AD1	GGGAGGTGGCTGACTGTGT		
MDV	MDV-F	GCCTTTTATACACAAGAGCCGAG	560 bp	Ding *et al* ., 2007
	MDV-R	TTTATCGCGGTTGTGGGTCATG		
REV	REV-F	CATACTGGAGCCAATGGTT	300 bp	Ji *et al* ., 2001
	REV-R	AATGTTGTAGCGAAGTACT		

[a]The exact size of PCR product depends on the ALV subgroup.

Table S6. Real-time PCR primers for IRF1, gp85, IFNβ and β-active.

Target gene	Primer pairs	Primer sequence (5' to 3')	Size of PCR product
IRF1	IRF1-F	GGCAGGAAGTGGAGGTGGAGA	132 bp
	IRF1-R	GCTGGTAGATGTCGTTGGTGCT	
ALV-J gp85	gp85-F	CACCTGGGCAAATAAGA	169 bp
	gp85-R	TTGGCTGGCTAAATCG	
IFNβ	IFNβ-F	CGTCACCAGGATGCCAACT	170 bp
	IFNβ-R	TGGAGGATGCGGAGGGT	
β-active	β-active-F	CCCCATGCCATCCTCCGTCTG	223 bp
	β-active-R	CCTCGGGGCACCTGAACCTCTC	
